# ﻿*Pestalotiopsis* (*Amphisphaeriales*, *Sporocadaceae*) species including six new taxa inhabiting pines from different climate zones in China

**DOI:** 10.3897/imafungus.16.151614

**Published:** 2025-06-10

**Authors:** Quan Chao Wang, Zhao Jie Zhan, Adil Sattar, Hao Nan Wang, Li Feng Zhou, Lori Eckhardt, Guo Qing Li, Fei Fei Liu, Hua Chao Xu, Xu Dong Zhou

**Affiliations:** 1 National Key Laboratory for Development and Utilization of Forest Food Resources, Zhejiang A&F University, Hangzhou 311300, China Zhejiang A&F University Hangzhou China; 2 College of Forestry and Landscape Architecture, Xinjiang Agricultural University, Urumqi 830052, China Xinjiang Agricultural University Urumqi China; 3 College of Forestry, Wildlife and Environment, Auburn University, 602 Duncan Drive, Ste 3301, Auburn, AL, USA Auburn University Auburn United States of America; 4 Research Institute of Fast-growing Trees (RIFT), Chinese Academy of Forestry (CAF), Zhanjiang 524000, Guangdong, China Research Institute of Fast-growing Trees (RIFT), Chinese Academy of Forestry (CAF) Zhanjiang China

**Keywords:** Phylogeny, pine needle disease, *Pinus*, taxonomy

## Abstract

Pine forest is important in China. However, its health has been increasingly threatened by pine needle blight caused by *Pestalotiopsis* species. Although several fungal species residing in this genus have been recorded, the diversity of *Pestalotiopsis* species inhabiting pine trees remains largely unresolved. In this study, a total of 209 diseased pine needle samples were collected from three provinces including Shandong, Zhejiang and Guangdong representing different climate zones in China. Subsequently, 100 isolates resembling *Pestalotiopsis* were obtained and 74 selected for characterisation, based on the internal transcribed spacer (ITS), translation elongation factor 1-alpha (*tef1-α*) and beta-tubulin (*tub2*) regions, as well as a combination of morphological characteristics. Ten *Pestalotiopsis* species were characterised including four known species (*Pes.clavata*, *Pes.disseminata*, *Pes.guangxiensis* and *Pes.lushanensis*) and six new to science, of which (*Pes.jiangmenensis*, *Pes.massoniana*, *Pes.ningboensis*, *Pes.shanweiensis*, *Pes.thunbergii* and *Pes.wenzhouensis*) are described here. This study further represents the first report of *Pes.clavata* and *Pes.guangxiensis* on *Pinus*. The results enhance our understanding and knowledge on the diversity of *Pestalotiopsis* inhabiting pines in China.

## ﻿Introduction

*Pinus (Pinaceae)* comprises a diverse group of trees, predominantly evergreen species and rare shrubs, encompassing more than 100 different species (Richardson 2000; Gernandt et al. 2005). Owing to their exceptional growth rate, robust adaptability and versatile applications, pine trees are widely distributed and planted worldwide (Rodríguez et al. 2022). Ecologically, they serve as effective wind-breakers and sand stabilisers promoting water conservation and air purification, thereby effectively mitigating soil erosion and fostering an optimal ecological milieu (Durrant et al. 2016; Mauri et al. 2016). Economically, pines are extensively utilised in construction engineering, furniture manufacturing, pulp and paper production, as well as wood processing industries, especially in China (Wieruszewski et al. 2023; Ding et al. 2024).

Exotic pests and pathogens have emerged as significant factors impeding the sustainable development of pine forest and industry. *Dendroctonusvalens*, an invasive pest originating from North America, has become a devastating exotic pest to pine forest in China, resulting in the loss of over 10 million *Pinustabuliformis* (Yan et al. 2005; Sun et al. 2013). Pine wilt disease mainly caused by *Bursapherenchusxylophilus* native to North America can infest over 50 *Pinus* species and has destroyed over 1.8 million ha of pine forest, representing the most notorious forest biotic threat in China (Ye and Wu 2022). *Pestalotiopsis*-like induced pine needle blight is a prevalent disease affecting various pine trees and it was first reported in 1974 in Sichuan Province (Qiu et al. 1980). Xu et al. (2017) conducted a systematic survey on *P.sylvestris* blight in northeast China, identifying *Pes.citri* as the causative agent. Further work by Chen et al. (2020) demonstrated the pathogenicity of *Pes.neglecta* to *P.sylvestris*. In 2024, *Pes.rosea* responsible for *P.thunbergii* blight (Han et al. 2024) and *Pes.jiangsuensis* for *P.massoniana* needle blight (Li et al. 2024) were reported. These findings indicate that *Pestalotiopsis*-induced pine needle blight may become an increasing threat to pine forests in China.

The taxonomy of pestalotioid genera is confusing and has undergone significant revisions. The genus *Pestalotia* was divided into *Truncatella* (4-celled), *Pestalotiopsis* (5-celled) and *Pestalotia* (6-celled), based on cell number of the conidial body by Steyaert (1949). *Monochaetia* was later classified as having five cells with a single apical and basal appendage (Guba 1956, 1961). The study of Maharachchikumbura et al. (2014) disclosed that ITS, *tef1-α* and *tub2* regions serve as reliable DNA barcoding markers differentiating *Pestalotiopsis* species, genera of *Neopestalotiopsis* and *Pseudopestalotiopsis*, and 24 species residing in the genus of *Pestalotiopsis* were thus described and resolved in the same study. Further, Liu et al. (2017) identified 15 *Pestalotiopsis* species from *Camellia*, Gu et al. (2021) described six novel *Pestalotiopsis* species from *Rhododendronsimsii* and Jiang et al. (2022c) reported 16 *Pestalotiopsis* species from fagaceous hosts. Recently, Razaghi et al. (2024) revised the family of *Sporocadaceae*, synonymising *Pes.kaki* with *Pes.menhaiensis* and *Pes.nanjingensis* with *Pes.sichuanensis* and described 14 *Pestalotiopsis* species. Currently, a total number of 437 *Pestalotiopsis* epithets have been included in Index Fungorum (http://www.indexfungorum.org/Names/Names.asp).

Pine needle blight caused by *Pestalotiopsis* primarily affects young pine forests, resulting in the withering of pine needles and, in severe cases, the complete defoliation and mortality of the tree’s canopy (Monteiro et al. 2022; Li et al. 2024). In this study, samples with typical pine needle blight symptoms were collected from different climate zones in China. Fungi were isolated and subjected to morphological and phylogenetic analyses. The objective of the study was to unveil the diversity of *Pestalotiopsis* species inhabiting pine trees from three provinces in different climatic zones in China.

## ﻿Materials and methods

### ﻿Sample collection and fungal isolation

**Figure 1.**
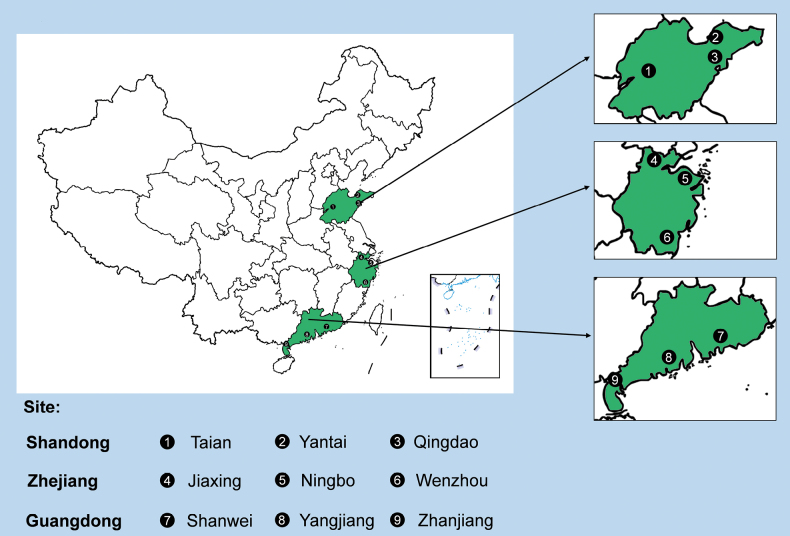
Distribution map of sample collection sites in China.

**Figure 2.**
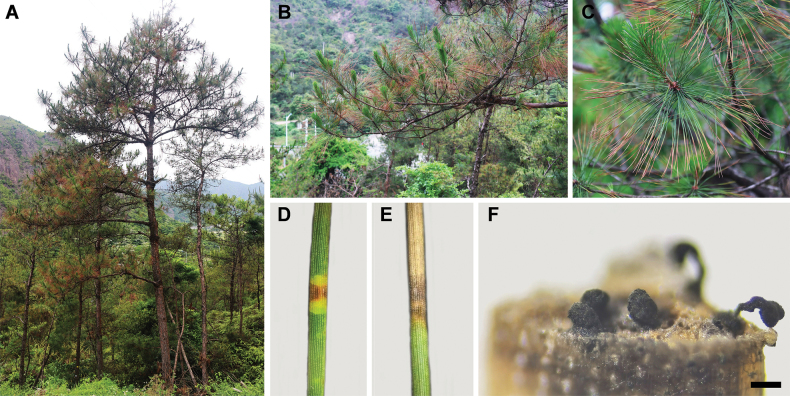
Disease symptoms on *Pinus* caused by species of *Pestalotiopsis*. **A–E** The red blight symptoms on pine needle; **F** appearance of conidiomata on host substrate.

From March 2023 to January 2024, disease surveys at the coastal pine protection forest were conducted in Shandong, Zhejiang and Guangdong Provinces in China (Fig. 1). During the initial stage of the disease, yellow spots emerge on the green needles, gradually turn into yellow or yellowish-brown segments, then light brownish-red and, finally, light grey or dark grey with light brown edges. Within the affected region, an oval shape will develop with a tear in the centre where black powder can be observed as the conidial disc of the pathogen (Fig. 2).

Strains were obtained by moist chamber and tissue culture methods from the symptomatic pine needles.

Moist chamber method: symptomatic tissues were incubated in moist Petri dishes at room temperature for 1–3 days to induce fungal sporulation (Fig. 2F). Conidial masses were transferred using sterile needles to 2% malt extract agar (MEA) (20 g malt extract and 20 g agar per litre of water; malt extract was obtained from the Beijing Shuangxuan and the agar from Beijing Solarbio Science & Technology Co., Ltd., Beijing, China).

Tissue culture method: the diseased/healthy interface was sectioned into 0.5 cm tissue blocks using a sterile blade. Subsequently, the tissue blocks were immersed in 70% ethanol for 30 seconds, followed by rinsing with a solution of 1.5% sodium hypochlorite for 90 seconds and three subsequent washes with sterile water, then the tissues were transferred on to the surface of MEA. The isolates were incubated at room temperature for 3–5 days; a single hyphal tip from each culture was transferred to a 2% fresh MEA plate and incubated at room temperature for 7–10 days to obtain pure cultures. The cultures and specimens were deposited in the China Forestry Culture Collection Center (CFCC; http://cfcc.caf.ac.cn/) and Fungarium of the Institute of Microbiology, Chinese Academy of Sciences (HMAS; https://nmdc.cn/fungarium/), respectively.

### ﻿DNA extraction, PCR, sequencing and phylogenetic analyses

The internal transcribed spacer (ITS) region of the obtained *Pestalotiopsis* strains was sequenced and analysed. Based on the sample locations and sequencing results, representative strains were selected for further sequencing of the translation elongation factor 1-alpha (*tef1-α*) and beta-tubulin (*tub2*) gene regions. Genomic DNA extraction was conducted on fresh mycelium growing on MEA at 25°C using the TreliefTM Plant Genomic DNA Kit according to the manufacturer’s instructions. Three distinct DNA regions (ITS, *tef1-α* and *tub2*) which can clearly distinguish between intraspecific and interspecific divergence of the *Pestalotiopsis* species were amplified (Maharachchikumbura et al. 2014; Jiang et al. 2022c; Razaghi et al. 2024).

Primers: ITS1/ITS4 were applied for the 5.8S nuclear ribosomal DNA gene with the two flanking internally transcribed spacer regions (White et al. 1990), EF1-728F/EF2 for the *tef1-α* gene (O’Donnell et al. 1998; Carbone and Kohn 1999) and Bt2a/Bt2b for the *tub2* gene (Glass and Donaldson 1995).

The PCR reaction mixture contained 25 µl of total volume, which consisted of 12 µl 2× High Fidelity PCR Master Mix (mixture of Super-Fidelity DNA Polymerase, MgCl_2_, dNTP Mix) (Sangon Biotech Co., Ltd., Shanghai, China), 1.5 µl of each forward and reverse primers, 9 µl ddH_2_O and 1 µl DNA. The PCR conditions were set as follows: an initial denaturation step of 5 min at 94°C, followed by 35 cycles of 30 s at 94°C, 50 s at 55°C (ITS) or 54°C (*tef1-α* and *tub2*) and 1 min at 72°C and a final elongation step of 10 min at 72°C. All PCR products were sequenced in both forward and reverse directions using the identical primers employed for PCR amplification. Sequence reactions were conducted by the Beijing Genomics Institute of Hangzhou, China. The nucleotide sequences were read and edited using Geneious v. 9.1.4 (Kearse et al. 2012). All sequences obtained in this study were submitted to GenBank (https://www.ncbi.nlm.nih.gov) (Table 1).

**Table 1.** Isolates of *Pestalotiopsis* sequenced and used for phylogenetic analyses in the current study.

Species	Culture no.	Substrate	Sampling site	Collectors	GPS	GenBank accession no.		
						ITS	*tub2*	*tef1-α*
*Pestalotiopsisclavata*	ZXD82	*Pinusmassoniana*	Wenzhou, Zhejiang	Q. C. Wang, G. Y. Cao	27°22'37.89"N, 120°36'48.81"E	PV259775	PV275170	PV275096
	ZXD99	*P.massoniana*	Wenzhou, Zhejiang	Q. C. Wang, G. Y. Cao	27°22'37.89"N, 120°36'48.81"E	PV259776	PV275171	PV275097
	ZXD100	*P.massoniana*	Wenzhou, Zhejiang	Q. C. Wang, G. Y. Cao	27°22'37.89"N, 120°36'48.81"E	PV259777	PV275172	PV275098
	ZXD106	*P.massoniana*	Wenzhou, Zhejiang	Q. C. Wang, G. Y. Cao	27°22'37.89"N, 120°36'48.81"E	PV259778	PV275173	PV275099
	ZXD124	*P.massoniana*	Wenzhou, Zhejiang	Q. C. Wang, G. Y. Cao	27°26'6.31"N, 120°29'40.31"E	PV259779	PV275174	PV275100
	ZXD565	*P.thunbergii*	Qingdao, Shandong	Q. C. Wang, G. Q. Li, F. F. Liu, R. L. Chang	36°25'2.69"N, 120°51'37.90"E	PV259780	PV275175	PV275101
	ZXD954	*P.massoniana*	Jiangmen, Guangdong	Q. C. Wang, G. Q. Li, F. F. Liu, Y. H. Liang	21°53'10.31"N, 112°57'0.87"E	PV259781	PV275176	PV275102
*Pes.disseminata*	ZXD34	*P.elliottii*	Ningbo, Zhejiang	Q. C. Wang, G. Y. Cao	29°5'44.31"N, 121°57'19.8"E	PV259782	PV275177	PV275103
	ZXD526	*P.thunbergii*	Qingdao, Shandong	Q. C. Wang, G. Q. Li, F. F. Liu, R. L. Chang	36°25'2.69"N, 120°51'37.90"E	PV259783	PV275178	PV275104
	ZXD529	*P.thunbergii*	Qingdao, Shandong	Q. C. Wang, G. Q. Li, F. F. Liu, R. L. Chang	36°25'2.69"N, 120°51'37.90"E	PV259784	PV275179	PV275105
	ZXD530	*P.thunbergii*	Qingdao, Shandong	Q. C. Wang, G. Q. Li, F. F. Liu, R. L. Chang	36°25'2.69"N, 120°51'37.90"E	PV259785	PV275180	PV275106
	ZXD532	*P.thunbergii*	Qingdao, Shandong	Q. C. Wang, G. Q. Li, F. F. Liu, R. L. Chang	36°25'2.69"N, 120°51'37.90"E	PV259786	PV275181	PV275107
	ZXD546	*P.thunbergii*	Qingdao, Shandong	Q. C. Wang, G. Q. Li, F. F. Liu, R. L. Chang	36°25'2.69"N, 120°51'37.90"E	PV259787	PV275182	PV275108
	ZXD552	*P.thunbergii*	Qingdao, Shandong	Q. C. Wang, G. Q. Li, F. F. Liu, R. L. Chang	36°25'2.69"N, 120°51'37.90"E	PV259788	PV275183	PV275109
	ZXD553	*P.thunbergii*	Qingdao, Shandong	Q. C. Wang, G. Q. Li, F. F. Liu, R. L. Chang	36°25'2.69"N, 120°51'37.90"E	PV259789	PV275184	PV275110
	ZXD577	*P.thunbergii*	Qingdao, Shandong	Q. C. Wang, G. Q. Li, F. F. Liu, R. L. Chang	36°25'2.69"N, 120°51'37.90"E	PV259790	PV275185	PV275111
	ZXD578	*P.thunbergii*	Qingdao, Shandong	Q. C. Wang, G. Q. Li, F. F. Liu, R. L. Chang	36°25'2.69"N, 120°51'37.90"E	PV259791	PV275186	PV275112
	ZXD579	*P.thunbergii*	Qingdao, Shandong	Q. C. Wang, G. Q. Li, F. F. Liu, R. L. Chang	36°25'2.69"N, 120°51'37.90"E	PV259792	PV275187	PV275113
	ZXD580	*P.thunbergii*	Qingdao, Shandong	Q. C. Wang, G. Q. Li, F. F. Liu, R. L. Chang	36°25'2.69"N, 120°51'37.90"E	PV259793	PV275188	PV275114
	ZXD582	*P.thunbergii*	Qingdao, Shandong	Q. C. Wang, G. Q. Li, F. F. Liu, R. L. Chang	36°25'2.69"N, 120°51'37.90"E	PV259794	PV275189	PV275115
	ZXD585	*P.thunbergii*	Qingdao, Shandong	Q. C. Wang, G. Q. Li, F. F. Liu, R. L. Chang	36°25'2.69"N, 120°51'37.90"E	PV259795	PV275190	PV275116
	ZXD586	*P.thunbergii*	Qingdao, Shandong	Q. C. Wang, G. Q. Li, F. F. Liu, R. L. Chang	36°25'2.69"N, 120°51'37.90"E	PV259796	PV275191	PV275117
	ZXD587	*P.thunbergii*	Qingdao, Shandong	Q. C. Wang, G. Q. Li, F. F. Liu, R. L. Chang	36°25'2.69"N, 120°51'37.90"E	PV259797	PV275192	PV275118
	ZXD588	*P.thunbergii*	Qingdao, Shandong	Q. C. Wang, G. Q. Li, F. F. Liu, R. L. Chang	36°25'2.69"N, 120°51'37.90"E	PV259798	PV275193	PV275119
*Pes.guangxiensis*	ZXD63	*P.massoniana*	Wenzhou, Zhejiang	Q. C. Wang, G. Q. Li, F. F. Liu, Y. H. Liang	27°22'37.89"N, 120°36'48.81"E	PV259799	PV275194	PV275120
	ZXD67	*P.massoniana*	Wenzhou, Zhejiang	Q. C. Wang, G. Q. Li, F. F. Liu, Y. H. Liang	27°22'37.89"N, 120°36'48.81"E	PV259800	PV275195	PV275121
	ZXD71	*P.massoniana*	Wenzhou, Zhejiang	Q. C. Wang, G. Y. Cao	27°22'37.89"N, 120°36'48.81"E	PV259801	PV275196	PV275122
	ZXD72	*P.massoniana*	Wenzhou, Zhejiang	Q. C. Wang, G. Y. Cao	27°22'37.89"N, 120°36'48.81"E	PV259802	PV275197	PV275123
	ZXD89	*P.massoniana*	Wenzhou, Zhejiang	Q. C. Wang, G. Y. Cao	27°22'37.89"N, 120°36'48.81"E	PV259803	PV275198	PV275124
*Pes.guangxiensis*	ZXD103	*P.massoniana*	Wenzhou, Zhejiang	Q. C. Wang, G. Y. Cao	27°22'37.89"N, 120°36'48.81"E	PV259804	PV275199	PV275125
	ZXD952	*P.massoniana*	Shanwei, Guangdong	Q. C. Wang, G. Y. Cao	21°53'10.31"N, 112°57'0.87"E	PV259805	PV275200	PV275126
	ZXD960	*P.massoniana*	Shanwei, Guangdong	Q. C. Wang, G. Y. Cao	21°53'10.31"N, 112°57'0.87"E	PV259806	PV275201	PV275127
	ZXD963	*P.massoniana*	Shanwei, Guangdong	Q. C. Wang, G. Y. Cao	21°53'10.31"N, 112°57'0.87"E	PV259807	PV275202	PV275128
	ZXD971	*P.massoniana*	Shanwei, Guangdong	Q. C. Wang, G. Y. Cao	21°53'10.31"N, 112°57'0.87"E	PV259808	PV275203	PV275129
	ZXD972	*P.massoniana*	Shanwei, Guangdong	Q. C. Wang, G. Q. Li, F. F. Liu, Y. H. Liang	21°53'10.31"N, 112°57'0.87"E	PV259809	PV275204	PV275130
***Pes.jiangmenensis***	**ZXD964 = CFCC 72595 T**	***P.massoniana***	**Jiangmen, Guangdong**	**Q. C. Wang, G. Q. Li, F. F. Liu, Y. H. Liang**	** 21°53'10.31"N, 112°57'0.87"E **	**PV259810**	**PV275205**	**PV275131**
	**ZXD965 = CFCC 72596**	***P.massoniana***	**Jiangmen, Guangdong**	**Q. C. Wang, G. Q. Li, F. F. Liu, Y. H. Liang**	** 21°53'10.31"N, 112°57'0.87"E **	**PV259811**	**PV275206**	**PV275132**
*Pes.lushanensis*	ZXD84	*P.massoniana*	Wenzhou, Zhejiang	Q. C. Wang, G. Q. Li, F. F. Liu, Y. H. Liang	27°22'37.89"N, 120°36'48.81"E	PV259812	PV275207	PV275133
	ZXD120	*P.massoniana*	Wenzhou, Zhejiang	Q. C. Wang, G. Q. Li, F. F. Liu, Y. H. Liang	27°26'6.31"N, 120°29'40.31"E	PV259813	PV275208	PV275134
	ZXD137	*P.massoniana*	Wenzhou, Zhejiang	Q. C. Wang, G. Q. Li, F. F. Liu, Y. H. Liang	27°26'6.31"N, 120°29'40.31"E	PV259814	PV275209	PV275135
	ZXD138	*P.massoniana*	Wenzhou, Zhejiang	Q. C. Wang, G. Q. Li, F. F. Liu, Y. H. Liang	27°26'6.31"N, 120°29'40.31"E	PV259815	PV275210	PV275136
	ZXD157	*P.massoniana*	Jiaxing, Zhejiang	Q. C. Wang, G. Y. Cao	30°36'10.01"N, 121°08'27.28"E	PV259816	PV275211	PV275137
	ZXD949	*P.massoniana*	Shanwei, Guangdong	Q. C. Wang, G. Y. Cao	21°53'10.31"N, 112°57'0.87"E	PV259817	PV275212	PV275138
	ZXD953	*P.massoniana*	Jiangmen, Guangdong	Q. C. Wang, G. Y. Cao	21°53'10.31"N, 112°57'0.87"E	PV259818	PV275213	PV275139
	ZXD969	*P.massoniana*	Jiangmen, Guangdong	Q. C. Wang, G. Y. Cao	21°53'10.31"N, 112°57'0.87"E	PV259819	PV275214	PV275140
***Pes.massoniana***	**ZXD955 = CFCC 72593 T**	***P.massoniana***	**Jiangmen, Guangdong**	**Q. C. Wang, G. Y. Cao**	** 21°53'10.31"N, 112°57'0.87"E **	**PV259820**	**PV275215**	**PV275141**
	**ZXD956 = CFCC 72594**	***P.massoniana***	**Jiangmen, Guangdong**	**Q. C. Wang, G. Q. Li, F. F. Liu, Y. H. Liang**	** 21°53'10.31"N, 112°57'0.87"E **	**PV259821**	**PV275216**	**PV275142**
***Pes.ningboensis***	**ZXD39 = CFCC 72585 T**	***P.elliottii***	**Ningbo, Zhejiang**	**Q. C. Wang, G. Q. Li, F. F. Liu, Y. H. Liang**	** 29°5'44.3076"N, 121°57'19.8"E **	**PV259822**	**PV275217**	**PV275143**
	**ZXD40 = CFCC 72586**	***P.elliottii***	**Ningbo, Zhejiang**	**Q. C. Wang, G. Q. Li, F. F. Liu, Y. H. Liang**	** 29°5'44.3076"N, 121°57'19.8"E **	**PV259823**	**PV275218**	**PV275144**
***Pes.shanweiensis***	**ZXD950 = CFCC 72591 T**	***P.massoniana***	**Shanwei, Guangdong**	**Q. C. Wang, G. Q. Li, F. F. Liu, Y. H. Liang**	** 21°53'10.31"N, 112°57'0.87"E **	**PV259824**	**PV275219**	**PV275145**
	**ZXD951 = CFCC 72592**	***P.massoniana***	**Shanwei, Guangdong**	**Q. C. Wang, G. Q. Li, F. F. Liu, Y. H. Liang**	** 21°53'10.31"N, 112°57'0.87"E **	**PV259825**	**PV275220**	**PV275146**
***Pes.thunbergii***	**ZXD204**	***P.massoniana***	**Jiaxing, Zhejiang**	**Q. C. Wang, G. Y. Cao**	** 30°36'10.01"N, 121°08'27.28"E **	**PV259826**	**PV275221**	**PV275147**
	**ZXD524**	***P.thunbergii***	**Qingdao, Shandong**	**Q. C. Wang, G. Q. Li, F. F. Liu, R. L. Chang**	** 36°25'2.69"N, 120°51'37.90"E **	**PV259827**	**PV275222**	**PV275148**
	**ZXD527**	***P.thunbergii***	**Qingdao, Shandong**	**Q. C. Wang, G. Q. Li, F. F. Liu, R. L. Chang**	** 36°25'2.69"N, 120°51'37.90"E **	**PV259828**	**PV275223**	**PV275149**
	**ZXD531**	***P.thunbergii***	**Qingdao, Shandong**	**Q. C. Wang, G. Q. Li, F. F. Liu, R. L. Chang**	** 36°25'2.69"N, 120°51'37.90"E **	**PV259829**	**PV275224**	**PV275150**
	**ZXD548**	***P.thunbergii***	**Qingdao, Shandong**	**Q. C. Wang, G. Q. Li, F. F. Liu, R. L. Chang**	** 36°25'2.69"N, 120°51'37.90"E **	**PV259830**	**PV275225**	**PV275151**
	**ZXD558**	***P.thunbergii***	**Qingdao, Shandong**	**Q. C. Wang, G. Q. Li, F. F. Liu, R. L. Chang**	** 36°25'2.69"N, 120°51'37.90"E **	**PV259831**	**PV275226**	**PV275152**
	**ZXD566**	***P.thunbergii***	**Qingdao, Shandong**	**Q. C. Wang, G. Q. Li, F. F. Liu, R. L. Chang**	** 36°25'2.69"N, 120°51'37.90"E **	**PV259832**	**PV275227**	**PV275153**
	**ZXD568**	***P.thunbergii***	**Qingdao, Shandong**	**Q. C. Wang, G. Q. Li, F. F. Liu, R. L. Chang**	** 36°25'2.69"N, 120°51'37.90"E **	**PV259833**	**PV275228**	**PV275154**
***Pes.thunbergii***	**ZXD569**	***P.thunbergii***	**Qingdao, Shandong**	**Q. C. Wang, G. Q. Li, F. F. Liu, R. L. Chang**	** 36°25'2.69"N, 120°51'37.90"E **	**PV259834**	**PV275229**	**PV275155**
	**ZXD575**	***P.thunbergii***	**Qingdao, Shandong**	**Q. C. Wang, G. Q. Li, F. F. Liu, R. L. Chang**	** 36°25'2.69"N, 120°51'37.90"E **	**PV259835**	**PV275230**	**PV275156**
	**ZXD576**	***P.thunbergii***	**Qingdao, Shandong**	**Q. C. Wang, G. Q. Li, F. F. Liu, R. L. Chang**	** 36°25'2.69"N, 120°51'37.90"E **	**PV259836**	**PV275231**	**PV275157**
	**ZXD581**	***P.thunbergii***	**Qingdao, Shandong**	**Q. C. Wang, G. Q. Li, F. F. Liu, R. L. Chang**	** 36°25'2.69"N, 120°51'37.90"E **	**PV259837**	**PV275232**	**PV275158**
	**ZXD583 = CFCC 72589 T**	***P.thunbergii***	**Qingdao, Shandong**	**Q. C. Wang, G. Q. Li, F. F. Liu, R. L. Chang**	** 36°25'2.69"N, 120°51'37.90"E **	**PV259838**	**PV275233**	**PV275159**
	**ZXD584 = CFCC 72590**	***P.thunbergii***	**Qingdao, Shandong**	**Q. C. Wang, G. Q. Li, F. F. Liu, R. L. Chang**	** 36°25'2.69"N, 120°51'37.90"E **	**PV259839**	**PV275234**	**PV275160**
***Pes.wenzhouensis***	**ZXD64 = CFCC 72587 T**	***P.massoniana***	**Wenzhou, Zhejiang**	**Q. C. Wang, G. Y. Cao**	** 27°22'37.89"N, 120°36'48.81"E **	**PV259840**	**PV275235**	**PV275161**
	**ZXD79**	***P.massoniana***	**Wenzhou, Zhejiang**	**Q. C. Wang, G. Y. Cao**	** 27°22'37.89"N, 120°36'48.81"E **	**PV259841**	**PV275236**	**PV275162**
	**ZXD86**	***P.massoniana***	**Wenzhou, Zhejiang**	**Q. C. Wang, G. Y. Cao**	** 27°22'37.89"N, 120°36'48.81"E **	**PV259842**	**PV275237**	**PV275163**
	**ZXD957**	***P.massoniana***	**Jiangmen, Guangdong**	**Q. C. Wang, G. Q. Li, F. F. Liu, Y. H. Liang**	** 21°53'10.31"N, 112°57'0.87"E **	**PV259843**	**PV275238**	**PV275164**
	**ZXD958**	***P.massoniana***	**Jiangmen, Guangdong**	**Q. C. Wang, G. Q. Li, F. F. Liu, Y. H. Liang**	** 21°53'10.31"N, 112°57'0.87"E **	**PV259844**	**PV275239**	**PV275165**
	**ZXD966 = CFCC 72588**	***P.massoniana***	**Jiangmen, Guangdong**	**Q. C. Wang, G. Q. Li, F. F. Liu, Y. H. Liang**	** 21°53'10.31"N, 112°57'0.87"E **	**PV259845**	**PV275240**	**PV275166**
	**ZXD970**	***P.massoniana***	**Jiangmen, Guangdong**	**Q. C. Wang, G. Q. Li, F. F. Liu, Y. H. Liang**	** 21°53'10.31"N, 112°57'0.87"E **	**PV259846**	**PV275241**	**PV275167**
	**ZXD973**	***P.massoniana***	**Jiangmen, Guangdong**	**Q. C. Wang, G. Q. Li, F. F. Liu, Y. H. Liang**	** 21°53'10.31"N, 112°57'0.87"E **	**PV259847**	**PV275242**	**PV275168**
	**ZXD974**	***P.massoniana***	**Jiangmen, Guangdong**	**Q. C. Wang, G. Q. Li, F. F. Liu, Y. H. Liang**	** 21°53'10.31"N, 112°57'0.87"E **	**PV259848**	**PV275243**	**PV275169**

The sequences generated in this study were subjected to phylogenetic analysis, comparing them with the sequences of type cultures of *Pestalotiopsis* species downloaded from GenBank (Table 2). Sequence alignment of ITS, *tef1-α* and *tub2* gene regions were performed in MAFFT online v. 7 (https://mafft.cbrc.jp/alignment/server/) with the alignment strategy FFT-NS-i (slow; interactive refinement method). Maximum Likelihood (ML) and Bayesian Inference (BI) analyses were performed separately using the PhyloSuite software (Zhang et al. 2020), with the concatenated ITS, *tef1-α* and *tub2* regions. The ModelFinder plug-in (Kalyaanamoorthy et al. 2017) implemented in PhyloSuite was utilised to determine TN+F+I+I+R4 and GTR+F+I+G4 as the ML and BI analyses evolutionary model, respectively. ML analysis was performed using the IQ-TREE plug-in version 1.6.8 (Ronquist and Huelsenbeck 2003) in PhyloSuite, with 5000 bootstrap replicates, 1000 interactions and a minimum correlation coefficient value of 0.99 generated. BI analysis was conducted using MrBayes plug-in (Ronquist and Huelsenbeck 2003) within PhyloSuite, “partition models” with 10,000,000 generations generated. The tree was edited using the software FigTree v.1.4.4. (Rambaut 2018) and MEGA v. 6.0.5 (Tamura et al. 2013). *Neopestalotiopsismagna* (MFLUCC 12-0652) was used as the outgroup taxon.

**Table 2.** Isolates of *Pestalotiopsis* from previous studies used in the phylogenetic analyses in the current study.

Species	Isolate	Host/substrate	Origin	GenBank accession no.			Reference
				ITS	tub2	tef1	
*Pes.abietis*	CFCC 53011*	*Abiesfargesii*	China	MK397013	MK622280	MK622277	Gu et al. (2021)
	CFCC 53012	*Abiesfargesii*	China	MK397014	MK622281	MK622278	Gu et al. (2021)
*Pes.adusta*	ICMP 6088*	Refrigerator door PVC gasket	Fiji	JX399006	JX399037	JX399070	Maharachchikumbura et al. (2012)
	MFLUCC 10-0146	*Syzygium* sp.	Thailand	JX399007	JX399038	JX399071	Maharachchikumbura et al. (2012)
*Pes.aggestorum*	LC6301*	*Camelliasinensis*	China	KX895015	KX895348	KX895234	Liu et al. (2017)
	LC8186	*Camelliasinensis*	China	KY464140	KY464160	KY464150	Liu et al. (2017)
*Pes.alloschemones*	CGMCC 3.23480 = LC13372*	*Alloschemoneoccidentalis*	China	OR247981	OR381056	OR361456	Razaghi et al. (2024)
	LC15841	*Alloschemoneoccidentalis*	China	OR247982	OR381057	OR361457	Razaghi et al. (2024)
*Pes.alpinicola*	HJAUP C1644.221*	*Alpiniazerumbet*	China	PP962274	PP952219	PP952249	Luo et al. (2024)
	HJAUP C1644.222	*Alpiniazerumbet*	China	PP962275	PP952220	PP952248	Luo et al. (2024)
*Pes.americana*	CBS 111576*	Leucospermumcunei×conocarpodendron	USA	MH553961	MH554620	MH554379	Liu et al. (2019)
*Pes.anacardiacearum*	IFRDCC 2397*	*Mangiferaindica*	China	KC247154	KC247155	KC247156	Maharachchikumbura et al. (2013b)
*Pes.anhuiensis*	CFCC 54791*	*Cyclobalanopsisglauca*	China	ON007028	ON005056	ON005045	Jiang et al. (2022c)
*Pes.appendiculata*	CGMCC 3.23550*	*Rhododendrondecorum*	China	OP082431	OP185516	OP185509	Gu et al. (2022)
*Pes.arceuthobii*	CBS 434.65*	*Arceuthobiumcampylopodum*	USA	KM199341	KM199427	KM199516	Maharachchikumbura et al. (2014)
*Pes.arengae*	CBS 331.92*	*Arengaundulatifolia*	Singapore	KM199340	KM199426	KM199515	Maharachchikumbura et al. (2014)
*Pes.australasiae*	CBS 114126*	*Knightia* sp.	New Zealand	KM199297	KM199409	KM199499	Maharachchikumbura et al. (2014)
	CBS 114141	*Protea* sp.	Australia	KM199298	KM199410	KM199501	Maharachchikumbura et al. (2014)
*Pes.australis*	CBS 111503	Proteaneriifolia×susannae	South Africa	KM199331	KM199382	KM199557	Maharachchikumbura et al. (2014)
	CBS 114193*	*Grevillea* sp.	Australia	KM199332	KM199383	KM199475	Maharachchikumbura et al. (2014)
*Pes.biappendiculata*	CGMCC 3.23487 = LC3574*	*Rhododendron* sp.	China	OR247984	OR381059	OR361459	Razaghi et al. (2024)
	LC4282	*Rhododendron* sp.	China	OR247990	OR381065	OR361465	Razaghi et al. (2024)
*Pes.biciliata*	CBS 124463*	Platanus×hispanica	Slovakia	KM199308	KM199399	KM199505	Maharachchikumbura et al. (2014)
	CBS 236.38	*Paeonia* sp.	ltaly	KM199309	KM199401	KM199506	Maharachchikumbura et al. (2014)
*Pes.brachiata*	LC2988*	*Camellia* sp.	China	KX894933	KX895265	KX895150	Liu et al. (2017)
	LC8188	*Camellia* sp.	China	KY464142	KY464162	KY464152	Liu et al. (2017)
*Pes.brassicae*	CBS 170.26*	*Brassicanapus*	New Zealand	KM199379	NA	KM199558	Maharachchikumbura et al. (2014)
*Pes.camelliae*	MFLUCC 12-0277*	*Camelliajaponica*	China	JX399010	JX399041	JX399074	Zhang et al. (2012b)
*Pes.camelliae-japonicae*	ZHKUCC 23-0826*	*Camelliajaponica*	China	OR258040	OR251483	OR251480	Dong et al. (2023)
	ZHKUCC 23-0827	*Camelliajaponica*	China	OR258041	OR251484	OR251481	Dong et al. (2023)
*Pes.camelliae-oleiferae*	CSUFTCC08*	*Camelliaeoleiferae*	China	OK493593	OK562368	OK507963	Li et al. (2021)
	CSUFTCC09	*Camelliaeoleiferae*	China	OK493594	OK562369	OK507964	Li et al. (2021)
*Pes.camelliicola*	HJAUP C1804.221*	*Camelliajaponica*	China	PP962357	PP952229	PP952236	Luo et al. (2024)
	HJAUP C1804.222	*Camelliajaponica*	China	PP962358	PP952230	PP952235	Luo et al. (2024)
*Pes.cangshanensis*	CGMCC 3.23544*	*Rhododendrondelavayi*	China	OP082426	OP185517	OP185510	Gu et al. (2022)
*Pes.castanopsidis*	CFCC 54430*	*Castanopsislamontii*	China	OK339732	OK358508	OK358493	Jiang et al. (2022c)
	CFCC 54305	*Castanopsishystrix*	China	OK339733	OK358509	OK358494	Jiang et al. (2022c)
*Pes.chamaeropis*	CBS 186.71*	*Chamaeropshumilis*	ltaly	KM199326	KM199391	KM199473	Maharachchikumbura et al. (2014)
	LC3619	*Camellia* sp.	China	KX894991	KX895322	KX895208	Liu et al. (2017)
*Pes.changjiangensis*	CFCC 54314*	*Castanopsistonkinensis*	China	OK339739	OK358515	OK358500	Jiang et al. (2022c)
	CFCC 54433	*Castanopsishainanensis*	China	OK339740	OK358516	OK358501	Jiang et al. (2022c)
*Pes.chaoyangensis*	CFCC 55549*	*Euonymusjaponicus*	China	OQ344763	OQ410584	OQ410582	Lin et al. (2023)
	CFCC 58805	*Euonymusjaponicus*	China	OQ344764	OQ410585	OQ410583	Lin et al. (2023)
*Pes.chiangmaiensis*	MFLUCC 22-0127*	*Phyllostachysedulis*	Thailand	OP497990	OP752137	OP753374	Sun et al. (2023)
*Pes.chiaroscuro*	BRIP 72970*	*Sporobolusnatalensis*	Australia	OK422510	OK423752	OK423753	Crous et al. (2022)
*Pes.chinensis*	MFLUCC 12-0273*	*Taxus* sp.	China	JX398995	NA	NA	Maharachchikumbura et al. (2012)
*Pes.clavata*	MFLUCC 12-0268*	*Buxus* sp.	China	JX398990	JX399025	JX399056	Maharachchikumbura et al. (2012)
	OP084	*Rhododendrondelavayi*	China	KC537803	KC537817	KC537810	Zhang et al. (2013)
*Pes.colombiensis*	CBS 118553*	*Eucalyptusurograndis*	Colombia	KM199307	KM199421	KM199488	Maharachchikumbura et al. (2014)
*Pes.cratoxyli*	CGMCC 3.23512 = LC8773*	*Cratoxylumcochinchinense*	China	OR248005	OR381080	OR361480	Razaghi et al. (2024)
	LC8780	*Cratoxylumcochinchinense*	China	OR248006	OR381081	OR361481	Razaghi et al. (2024)
*Pes.cyclobalanopsidis*	CFCC 54328*	*Cyclobalanopsisglauca*	China	OK339735	OK358511	OK358496	Jiang et al. (2022c)
	CFCC 55891	*Cyclobalanopsisglauca*	China	OK339736	OK358512	OK358497	Jiang et al. (2022c)
*Pes.cyclosora*	HJAUP C1724.221*	*Cyclosorusinterruptus*	China	PP962279	PP952221	PP952247	Luo et al. (2024)
	HJAUP C1724.222	*Cyclosorusinterruptus*	China	PP962280	PP952222	PP952246	Luo et al. (2024)
*Pes.daliensis*	CGMCC 3.23548*	*Rhododendrondecorum*	China	OP082429	OP185518	OP185511	Gu et al. (2022)
*Pes.dianellae*	CBS 143421*	*Dianella* sp.	Australia	NR156664	MG386164	NA	Crous et al. (2017)
*Pes.digitalis*	ICMP 5434*	*Digitalispurpurea*	New Zealand	KP781879	KP781883	NA	Liu et al. (2015)
*Pes.dilucida*	LC3232*	*Camelliasinensis*	China	KX894961	KX895293	KX895178	Liu et al. (2017)
	LC8184	*Camelliasinensis*	China	KY464138	KY464158	KY464148	Liu et al. (2017)
*Pes.diploclisiae*	CBS 115449	*Psychotriatutcheri*	China	KM199314	KM199416	KM199485	Maharachchikumbura et al. (2014)
	CBS 115587*	*Diploclisiaglaucescens*	China	KM199320	KM199419	KM199486	Maharachchikumbura et al. (2014)
*Pes.disseminata*	CBS 143904	*Perseaamericana*	New Zealand	MH554152	MH554825	MH554587	Liu et al. (2019)
	CBS 118552	*Eucalyptusbotryoides*	New Zealand	MH553986	MH554652	MH554410	Liu et al. (2019)
	MEAN 1165	*Pinuspinea*, blighted shoot	Portugal	MT374687	MT374712	MT374699	Silva et al. (2020)
	MEAN 1166	*Pinuspinea*, blighted shoot	Portugal	MT374688	MT374713	MT374700	Silva et al. (2020)
*Pes.diversiseta*	MFLUCC 12-0287*	*Rhododendron* sp.	China	JX399009	JX399040	JX399073	Maharachchikumbura et al. (2012)
*Pes.doitungensis*	MFLUCC 14-0115*	*Dendrobium* sp.	Thailand	MK993574	MK975837	MK975832	Ma et al. (2019)
*Pes.dracaenae*	HGUP 4037*	*Dracaenafragrans*	China	MT596515	MT598645	MT598644	Ariyawansa et al. (2015)
*Pes.dracaenicola*	MFLUCC 18-0913*	*Dracaena* sp.	Thailand	MN962731	MN962733	MN962732	Chaiwan et al. (2020)
*Pes.dracontomelon*	MFLUCC 10-0149*	*Dracontomelondao*	Thailand	NR168755	NA	KP781880	Liu et al. (2015)
*Pes.endophytica*	MFLUCC 18-0932*	*Magnoliagarrettii*	Thailand	MW263946	NA	MW417119	de Silva et al. (2021)
*Pes.ericacearum*	IFRDCC 2439*	*Rhododendrondelavayi*	China	KC537807	KC537821	KC537814	Zhang et al. (2013)
*Pes.eriobotryae*	HJAUP C1742.221*	*Eriobotryajaponica*	China	PP962289	PP952227	PP952238	Luo et al. (2024)
	HJAUP C1742.222	*Eriobotryajaponica*	China	PP962291	PP952228	PP952237	Luo et al. (2024)
*Pes.etonensis*	BRIP 66615*	*Sporobolusjacquemontii*	Australia	MK966339	MK977634	MK977635	Crous et al. (2020)
*Pes.exudata*	CGMCC 3.23488 = LC3582*	*Aucubajaponica*	China	OR247985	OR381060	OR361460	Razaghi et al. (2024)
	LC15850	*Aucubajaponica*	China	OR247986	OR381061	OR361461	Razaghi et al. (2024)
*Pes.ficicola*	SAUCC230046*	*Ficusmicrocarpa*	China	OQ691974	OQ718749	OQ718691	Zhang et al. (2023)
	SAUCC230042	*Ficusmicrocarpa*	China	OQ691972	OQ718747	OQ718689	Zhang et al. (2023)
*Pes.ficicrescens*	GUCC 21556	*Ficustikoua*	China	MZ477311	MZ868301	MZ868328	Hyde et al. (2023)
	CGMCC 3.23471 = LC12337*	*Oleaceae*	China	OR247980	OR381055	OR361455	Razaghi et al. (2024)
*Pes.foliicola*	CFCC 54440*	*Castanopsisfaberi*	China	ON007029	ON005057	ON005046	Jiang et al. (2022c)
	CFCC 57359	*Castanopsisfaberi*	China	ON007030	ON005058	ON005047	Jiang et al. (2022c)
*Pes.formosana*	NTUCC 17-009*	*Poaceae* sp.	China	MH809381	MH809385	MH809389	Ariyawansa and Hyde (2018)
*Pes.furcata*	MFLUCC 12-0054	*Cameliasinensis*	Thailand	JQ683724	JQ683708	JQ683740	Maharachchikumbura et al. (2013a)
	LC6691*	*Cameliasinensis*	China	KX895030	KX895363	KX895248	Liu et al. (2017)
*Pes.fusiformis*	CGMCC 3.23495 = LC4365	*Rhododendron* sp.	China	OR247995	OR381070	OR361470	Razaghi et al. (2024)
	LC15852	*Rhododendron* sp.	China	OR247996	OR381071	OR361471	Razaghi et al. (2024)
*Pes.fusoidea*	CGMCC 3.23545*	*Rhododendrondelavayi*	China	OP082427	OP185519	OP185512	Gu et al. (2022)
*Pes.ganzhouensis*	CGMCC 3.23489 = LC3629*	*Cinnamomumcamphora*	China	OR247987	OR381062	OR361462	Razaghi et al. (2024)
	LC5089	*Cinnamomumcamphora*	China	OR247998	OR381073	R361473	Razaghi et al. (2024)
*Pes.gardeniae*	HJAUP C1729.221*	*Gardeniajasminoides*	China	PP962285	PP952225	PP952241	Luo et al. (2024)
	HJAUP C1729.222	*Gardeniajasminoides*	China	PP962286	PP952226	PP952240	Luo et al. (2024)
*Pes.gaultheriae*	IFRD 411-014*	*Gaultheriaforrestii*	China	KC537805	KC537819	KC537812	Zhang et al. (2013)
*Pes.gibbosa*	NOF 3175*	*Gaultheriashallon*	Canada	LC311589	LC311590	LC311591	Watanabe et al. (2018)
*Pes.grandis-urophylla*	E-72-02	*Eucalyptus* sp.	Brazil	KU926708	KU926716	KU926712	Carvalho et al. (2019)
	E-72-03	*Eucalyptus* sp.	Brazil	KU926709	KU926717	KU926713	Carvalho et al. (2019)
*Pes.grevilleae*	CBS 114127*	*Grevillea* sp.	Australia	KM199300	KM199407	KM199504	Maharachchikumbura et al. (2014)
*Pes.guangdongensis*	ZHKUCC 22-0016*	*Arengapinnata*	China	ON180762	ON221548	ON221520	Xiong et al. (2022)
	ZHKUCC 22-0017	*Arengapinnata*	China	ON180763	ON221549	ON221521	Xiong et al. (2022)
*Pes.guangxiensis*	CFCC 54308*	*Quercusgriffithii*	China	OK339737	OK358513	OK358498	Jiang et al. (2022c)
	CFCC 54300	*Quercusgriffithii*	China	OK339738	OK358514	OK358499	Jiang et al. (2022c)
*Pes.guiyangensis*	CFCC 70626*	*Eriobotryajaponica*	China	PP784740	PP842617	PP842629	Zhang et al. (2024)
	CFCC 70630	*Rohdeajaponica*	China	PP784741	PP842618	PP842630	Zhang et al. (2024)
*Pes.guizhouensis*	CFCC 54803*	*Cyclobalanopsisglauca*	China	ON007035	ON005063	ON005052	Jiang et al. (2022c)
	CFCC 57364	*cyclobalanopsisglauca*	China	ON007036	ON005064	ON005053	Jiang et al. (2022c)
*Pes.hainanensis*	PSHI2004Endo166*	*Podocarpusmacrophyllus*	China	DQ334863	DQ137861	NA	Liu et al. (2017)
*Pes.hawaiiensis*	CBS 114491*	*Leucospermum* sp.	USA	KM199339	KM199428	KM199514	Maharachchikumbura et al. (2014)
*Pes.hederae*	HJAUP C1638.221*	*Hederahelix*	China	PP962270	PP952234	PP952252	Luo et al. (2024)
	HJAUP C1638.222	*Hederahelix*	China	PP962271	PP952216	NA	Luo et al. (2024)
*Pes.hispanica*	CBS 115.391*	*Protea* sp.	Spain	MH553981	MH554640	MH554399	Liu et al. (2019)
*Pes.hollandica*	CBS 265.33*	*Sciadopitysverticilata*	Netherlands	KM199328	KM199388	KM199481	Maharachchikumbura et al. (2014)
*Pes.humus*	CBS 336.97*	Soil	Papua New Guinea	KM199317	KM199420	KM199484	Maharachchikumbura et al. (2014)
*Pes.hunanensis*	CSUFTCC15*	*Camelliaoleifera*	China	OK493599	OK562374	OK507969	Li et al. (2021)
	CSUFTCC18	*Camelliaoleifera*	China	OK493600	OK562375	OK507970	Li et al. (2021)
*Pes.hydei*	MFLUCC 20-0135*	*Litseaelliptica*	Thailand	NR172003	MW251112	MW251113	Huanaluek et al. (2021)
*Pes.iberica*	CAA 1004*	*Pinusradiata*	Spain	MW732248	MW759035	MW759038	Monteiro et al. (2022)
	CAA 1005	*Pinussylvestris*	Spain	MW732250	MW759034	MW759037	Monteiro et al. (2022)
*Pes.inflexa*	MFLUCC 12-0270*	Unidentified tree	China	JX399008	JX399039	JX399072	Maharachchikumbura et al. (2012)
*Pes.intermedia*	MFLUCC 12-0259*	Unidentified tree	China	JX398993	JX399028	JX399059	Maharachchikumbura et al. (2012)
*Pes.italiana*	MFLUCC 12-0657*	*Cupressusglabra*	ltaly	KP781878	KP781882	KP781881	Liu et al. (2015)
*Pes.jesteri*	CBS 109350*	*Fragraeabodenii*	Papua New Guinea	KM199380	KM199468	OR380983	Maharachchikumbura et al. (2014)
*Pes.jiangsuensis*	CFCC 59538*	*Pinusmassoniana*	China	OR533577	OR539191	OR539186	Li et al. (2024)
	CFCC 59539	*Pinusmassoniana*	China	OR533578	OR539192	OR539187	Li et al. (2024)
*Pes.jiangxiensis*	LC4399*	*Camellia* sp.	China	KX895009	KX895341	KX895227	Liu et al. (2017)
*Pes.jinchanghensis*	LC6636*	*Camelliasinensis*	China	KX895028	KX895361	KX895247	Liu et al. (2017)
	LC8190	*Camelliasinensis*	China	KY464144	KY464164	KY464154	Liu et al. (2017)
*Pes.kandelicola*	NCYU 19-0354	*Kandeliacandel*	China	MT560723	MT563100	MT563102	Hyde et al. (2020)
	NCYU 19-0355*	*Kandeliacandel*	China	MT560722	MT563099	MT563101	Hyde et al. (2020)
*Pes.kenyana*	CBS 442.67*	*Coffea* sp.	Kenya	KM199302	KM199395	KM199502	Maharachchikumbura et al. (2014)
	LC6633	*Camelliasinensis*	China	KX895027	KX895360	KX895246	Maharachchikumbura et al. (2014)
*Pes.knightiae*	CBS 111963	*Knightia* sp.	New Zealand	KM199311	KM199406	KM199495	Maharachchikumbura et al. (2014)
	CBS 114138*	*Knightia* sp.	New Zealand	KM199310	KM199408	KM199497	Maharachchikumbura et al. (2014)
*Pes.krabiensis*	MFLUCC 16-0260*	*Pandanus* sp.	Thailand	MH388360	MH412722	MH388395	Tibpromma et al. (2018)
*Pes.kunmingensis*	PSHI2002Endo766*	*Podocarpusmacrophyllus*	China	AY373376	DQ333576	NA	Wei and Xu (2004)
*Pes.leucadendri*	CBS 121417*	*leucadendron* sp.	South Africa	MH553987	MH554654	MH554412	Liu et al. (2019)
*Pes.leucospermi*	CBS 114489*	*Leucospermum* cv. ‘Pink Ice’	USA	MH553978	MH554637	MH554396	Liu et al. (2019)
*Pes.licualacola*	HGUP 4057*	*Licualagrandis*	China	KC492509	KC481683	KC481684	Geng et al. (2013)
*Pes.lijiangensis*	CFCC 50738*	Castanopsiscarlesiivar.spinulosa	China	KU860520	KU844184	KU844185	Zhou et al. (2018)
	CFCC 50739	Castanopsiscarlesiivar.spinulosa	China	MH880834	MH880835	MH880836	Zhou et al. (2018)
*Pes.linearis*	MFLUCC 12-0271*	*Trachelospermum* sp.	China	JX398992	JX399027	JX399058	Maharachchikumbura et al. (2012)
*Pes.linguae*	ZHKUCC 22-0159*	*Pyrrosialingua*	China	OP094104	OP186108	OP186110	Li et al. (2023)
	ZHKUCC 22-0160	*Pyrrosialingua*	China	OP094103	OP186107	OP186109	Li et al. (2023)
*Pes.lithocarpi*	CFCC 55100*	*Lithocarpuschiungchungensis*	China	OK339742	OK358518	OK358503	Jiang et al. (2022c)
	CFCC 55893	*Lithocarpuschiungchungensis*	China	OK339743	OK358519	OK358504	Jiang et al. (2022c)
*Pes.lobata*	CGMCC 3.23467 = LC1102*	*Lithocarpusglaber*	China	OR247976	OR381051	OR361451	Razaghi et al. (2024)
	LC15843	*Lithocarpusglaber*	China	OR247977	OR381052	OR361452	Razaghi et al. (2024)
*Pes.loeiana*	MFLUCC 22-0123*	Unidentified plant	Thailand	OP497988	OP713769	OP737881	Sun et al. (2023)
*Pes.longiappendiculata*	LC3013*	*Camelliasinensis*	China	KX894939	KX895271	KX895156	Liu et al. (2017)
*Pes.lushanensis*	LC4344*	*Camellia* sp.	China	KX895005	KX895337	KX895223	Liu et al. (2017)
	LC8182	*Camellia* sp.	China	KY464136	KY464156	KY464146	Liu et al. (2017)
*Pes.macadamiae*	BRIP 63738b*	*Macadamiaintegrifolia*	Australia	KX186588	KX186680	KX186621	Akinsanmi et al. (2017)
	BRIP 63739b	*Macadamiaintegrifolia*	Australia	KX186587	KX186679	KX186620	Akinsanmi et al. (2017)
*Pes.machili*	CGMCC 3.23511 = LC8736*	*Machilus* sp.	China	OR248003	OR381078	OR361478	Razaghi et al. (2024)
*Pes.machiliana*	HJAUP C1790.221*	*Machiluspauhoi*	China	PP962355	PP952214	PP952253	Luo et al. (2024)
	HJAUP C1790.222	*Machiluspauhoi*	China	PP962356	PP952215	PP952254	Luo et al. (2024)
*Pes.malayana*	CBS 102220*	*Macarangatriloba*	Malaysia	KM199306	KM199411	KM199482	Maharachchikumbura et al. (2014)
*Pes.mangifericola*	HJAUP C1639.221*	*Mangiferaindica*	China	PP962272	PP952217	PP952251	Luo et al. (2024)
	HJAUP C1639.222	*Mangiferaindica*	China	PP962273	PP952218	PP952250	Luo et al. (2024)
*Pes.manyueyuanani*	NTUPPMCC 18-165*	*Ophocordyceps* sp.	Taiwan	OR125060	OR126306	OR126313	Hsu et al. (2024)
	NTUPPMCC 22-012	*Ophocordyceps* sp.	Taiwan	OR125061	OR126307	OR126314	Hsu et al. (2024)
*Pes.menhaiensis*	CGMCC 3.18250*	*Camelliasinensis*	China	KU252272	KU252488	KU252401	Wang et al. (2019)
	KNU-PT-1804*	*Diospyroskaki*	Korea	LC552953	LC552954	LC553555	Das et al. (2021), Razaghi et al. (2024)
*Pes.monochaeta*	CBS 144.97*	*Quercusrobur*	Netherlands	KM199327	KM199386	KM199479	Maharachchikumbura et al. (2014)
	CBS 440.83	*Taxusbaccata*	Netherlands	KM199329	KM199387	KM199480	Maharachchikumbura et al. (2014)
*Pes.montellica*	MFLUCC 12-0279*	Dead plant material	China	JX399012	JX399043	JX399076	Ariyawansa et al. (2015)
*Pes.multiappendiculata*	CGMCC 3.23514 = LC2911 = LF118*	NA	China	OR248008	OR381083	OR361483	Razaghi et al. (2024)
*Pes.multicolor*	CFCC 59981*	*Taxuschinensis*	China	OQ626676	OQ714336	OQ714341	Wang et al. (2024)
	CFCC 59982	*Taxuschinensis*	China	OQ771896	OQ779488	OQ779483	Wang et al. (2024)
*Pes.nanningensis*	CSUFTCC10*	*Camelliaoleifera*	China	OK493596	OK562371	OK507966	Li et al. (2021)
*Pes.neglecta*	MAFF 239735*	*Quercusmyrsinaefolia*	Japan	AB482220	LC311599	LC311600	Watanabe et al. (2018)
*Pes.neolitseae*	NTUCC 17-011*	*Neolitseavillosa*	China	MH809383	MH809387	MH809391	Ariyawansa and Hyde (2018)
	CFCC 54590	*Lithocarpusamygdalifolius*	China	OK339744	OK358520	OK358505	Jiang et al. (2022c)
*Pes.novae-hollandiae*	CBS 130973*	*Banksiagrandis*	Australia	KM199337	KM199425	KM199511	Maharachchikumbura et al. (2014)
*Pes.olivacea*	SY17A	NA	NA	EF055215	EF055251	NA	Zhou et al. (2018)
*Pes.oryzae*	CBS 111522	*Relopea* sp.	USA	KM199294	KM199394	KM199493	Maharachchikumbura et al. (2014)
	CBS 171.26	NA	ltaly	KM199304	KM199397	KM199494	Maharachchikumbura et al. (2014)
	CBS 353.69*	*Oryzasativa*	Denmark	KM199299	KM199398	KM199496	Maharachchikumbura et al. (2014)
*Pes.pallidotheae*	MAFF 240993*	*Pierisjaponica*	Japan	NR111022	LC311584	LC311585	Watanabe et al. (2010)
*Pes.pandanicola*	MFLUCC 16-0255*	*Pandanus* sp.	Thailand	MH388361	MH412723	MH388396	Tibpromma et al. (2018)
*Pes.papuana*	CBS 331.96*	Coastal soil	Papua New Guinea	KM199321	KM199413	KM199491	Maharachchikumbura et al. (2014)
	CBS 887.96	*Cocosnucifera*	Papua New Guinea	KM199318	KM199415	KM199492	Maharachchikumbura et al. (2014)
*Pes.parva*	CBS 265.37	*Delonixregia*	NA	KM199312	KM199404	KM199508	Maharachchikumbura et al. (2014)
	CBS 278.35*	*Delonixregia*	NA	KM199313	KM199405	KM199509	Maharachchikumbura et al. (2014)
*Pes.phoebes*	SAUCC230093*	*Phoebezhennan*	China	OQ692028	OQ718803	OQ718745	Zhang et al. (2023)
	SAUCC230092	*Phoebezhennan*	China	OQ692027	OQ718802	OQ718744	Zhang et al. (2023)
*Pes.photinicola*	GZCC16-0028*	*Photiniaserrulata*	China	KY092404	KY047663	KY047662	Chen et al. (2017)
*Pes.pini*	MEAN 1092	*Pinuspinea*	Portugal	MT374680	MT374705	MT374693	Silva et al. (2020)
	MEAN 1094*	*Pinuspinea*	Portugal	MT374681	MT374706	MT374694	Silva et al. (2020)
*Pes.pinicola*	KUMCC 19-0183*	*Pinusarmandii*	China	MN412636	MN417507	MN417509	Tibpromma et al. (2019)
*Pes.piraubensis*	COAD 2165*	*Psidiumguajava*	Brazil	MH627381	MH643773	MH643774	Jayawardena et al. (2022)
*Pes.portugalica*	CBS 393.48*	NA	Portugal	KM199335	KM199422	KM199510	Maharachchikumbura et al. (2014)
*Pes.pruni*	CGMCC 3.23507 = LC8252*	*Prunuscerasoides*	China	OR248001	OR381076	OR361476	Razaghi et al. (2024)
	LC15860	*Prunuscerasoides*	China	OR248002	OR381077	OR361477	Razaghi et al. (2024)
*Pes.pyrrosiae-linguae*	ZHKUCC 23-0807*	*Pyrrosialingua*	China	OR199902	OR259258	OR259260	Dong et al. (2023)
	ZHKUCC 23-0808	*Pyrrosialingua*	China	OR199903	OR259259	OR259261	Dong et al. (2023)
*Pes.rhizophorae*	MFLUCC 17-0416*	*Rhizophoramucronata*	Thailand	MK764283	MK764349	MK764327	Norphanphoun et al. (2019)
*Pes.rhododendri*	IFRDCC 2399*	*Rhododendronsinogrande*	China	KC537804	KC537818	KC537811	Norphanphoun et al. (2019)
*Pes.rhodomyrtus*	LC4458	*Camelliasinensis*	China	KX895010	KX895342	KX895228	Norphanphoun et al. (2019)
	HGUP 4230*	*Rhodomyrtustomentosa*	China	KF412648	KF412642	KF412645	Song et al. (2013)
*Pes.rosarioides*	CGMCC 3.23549*	*Rhododendrondecorum*	China	OP082430	OP185520	OP185513	Gu et al. (2022)
*Pes.rosea*	MFLUCC 12-0258*	*Pinus* sp.	China	JX399005	JX399036	JX399069	Maharachchikumbura et al. (2012)
*Pes.rubrae*	CGMCC 3.23499 = LC4567*	*Quercusrubra*	China	OR247997	OR381072	OR361472	Razaghi et al. (2024)
	LC8233	*Plagiogyriaglauca*	China	OR248000	OR381075	OR361475	Razaghi et al. (2024)
*Pes.sabal*	ZHKUCC 22-0031	*Sabalmexicana*	China	ON180769	ON221555	ON221527	Xiong et al. (2022)
	ZHKUCC 22-0035*	*Sabalmexicana*	China	ON180775	ON221561	ON221533	Xiong et al. (2022)
*Pes.schisandrae*	CFCC 59550	*Schisandrasphenanthera*	China	OR775411	OR766014	OR766002	Yuan et al. (2024)
	CFCC 59551*	*Schisandrasphenanthera*	China	OR775412	OR766015	OR766003	Yuan et al. (2024)
*Pes.scoparia*	CBS 176.25*	*Chamaecyparis* sp.	China	KM199330	KM199393	KM199478	Maharachchikumbura et al. (2012)
*Pes.sequoiae*	MFLUCC 13-0399*	*Sequoiasempervirens*	ltaly	KX572339	NA	NA	Maharachchikumbura et al. (2012)
*Pes.shaanxiensis*	CFCC 54958*	*Quercusvariabilis*	China	ON007026	ON005054	ON005043	Jiang et al. (2022c)
	CFCC 57356	*Quercusvariabilis*	China	ON007027	ON005055	ON005044	Jiang et al. (2022c)
*Pes.shoreae*	MFLUCC 12-0314*	*Shoreaobtusa*	Thailand	KJ503811	KJ503814	KJ503817	Song et al. (2014)
*Pes.sichuanensis*	CGMCC 3.18244*	*Camelliasinensis*	China	KX146689	KX146807	KX146748	Wang et al. (2019)
	CFCC 53882	*Quercusaliena*	China	OM746295	OM839968	OM840067	Li et al. (2021)
	CSUFTCC16	*Camelliaoleifera*	China	OK493602	OK562377	OK507972	Li et al. (2021)
*Pes.silvicola*	CFCC 55296*	*Cyclobalanopsiskerrii*	China	ON007032	ON005060	ON005049	Jiang et al. (2022c)
	CFCC 54915	*Cyclobalanopsiskerrii*	China	ON007033	ON005061	ON005050	Jiang et al. (2022c)
*Pes.smilacicola*	MFLUCC 22-0125*	*Smilax* sp.	Thailand	OP497991	OP762673	OP753376	Sun et al. (2023)
	MFLUCC 22-0124	*Smilax* sp.	Thailand	OP497989	OP762674	OP737879	Sun et al. (2023), Razaghi et al. (2024)
*Pes.sonneratiae*	CFCC 57394*	*Sonneratiaapetala*	China	ON114184	ON086816	ON086812	Jiang et al. (2022b)
	CFCC 57395	*Sonneratiaapetala*	China	ON114185	ON086817	ON086813	Jiang et al. (2022b)
*Pes.spatholobi*	SAUCC231201*	*Spatholobussuberectus*	China	OQ692023	OQ718798	OQ718740	Zhang et al. (2023)
	SAUCC231203	*Spatholobussuberectus*	China	OQ692024	OQ718799	OQ718741	Zhang et al. (2023)
*Pes.spathulata*	CBS 356.86*	*Gevuinaavellana*	Chile	KM199338	KM199423	KM199513	Maharachchikumbura et al. (2014)
*Pes.spathuliappendiculata*	CBS 144035*	*Phoenixcanariensis*	Australia	MH554172	MH554845	MH554607	Liu et al. (2019)
*Pes.suae*	CGMCC 3.23546*	*Rhododendrondelavayi*	China	OP082428	OP185521	OP185514	Gu et al. (2022)
*Pes.taxicola*	CFCC 59976*	*Taxuschinensis*	China	OQ626673	OQ714333	OQ714338	Wang et al. (2024)
	CFCC 59978	*Taxuschinensis*	China	OQ771893	OQ779485	OQ779480	Wang et al. (2024)
*Pes.telopeae*	CBS 113606	*Telopea* sp.	Australia	KM199295	KM199402	KM199498	Maharachchikumbura et al. (2014)
	CBS 114161*	*Telopea* sp.	Australia	KM199296	KM199403	KM199500	Maharachchikumbura et al. (2014)
*Pes.terricola*	CBS 141.69*	Soil	Pacific Islands	MH554004	MH554680	MH554438	Liu et al. (2019)
*Pes.thailandica*	MFLUCC 17-1616*	*Rhizophoramucronata*	Thailand	NR164471	MK764352	MK764330	Maharachchikumbura et al. (2014)
	MFLUCC 17-1617	*Rhizophoramucronata*	Thailand	MK764285	MK764351	MK764329	Maharachchikumbura et al. (2014)
*Pes.trachicarpicola*	OP143	*Podocarpusmacrophyllus*	China	KC537809	KC537823	KC537816	Zhang et al. (2012a)
	LC4523	*Camelliasinensis*	China	KX895011	KX895344	KX895230	Liu et al. (2017)
	OP068*	*Trachycarpusfortunei*	China	JQ845947	JQ845945	JQ845946	Zhang et al. (2012a)
*Pes.tumida*	CFCC 55158*	*Rosachinensis*	China	OK560610	OM158174	OL814524	Peng et al. (2022)
*Pes.unicolor*	MFLUCC 12-0275	Unidentified tree	China	JX398998	JX399029	JX399063	Maharachchikumbura et al. (2012)
	MFLUCC 12-0276*	*Rhododendron* sp.	China	JX398999	JX399030	NA	Maharachchikumbura et al. (2012)
*Pes.verruculosa*	MFLUCC 12-0274*	*Rhododendron* sp.	China	JX398996	NA	JX399061	Maharachchikumbura et al. (2012)
*Pes.wulichongensis*	CGMCC 3.23469 = LC11341*	*Poaceae*	China	OR247978	OR381053	OR361453	Razaghi et al. (2024)
	LC15846	*Poaceae*	China	OR247979	OR381054	OR361454	Razaghi et al. (2024)
*Pes.xuefengensis*	HJHB1*	*Polygonatumcyrtonema*	China	OQ711603	OQ737677	OQ737676	Sun et al. (2024)
	HJHB5	*Polygonatumcyrtonema*	China	OQ746334	OQ772277	OQ772275	Sun et al. (2024)
*Pes.yanglingensis*	LC3412	*Camelliasinensis*	China	KX894980	KX895312	KX895197	Liu et al. (2017)
	LC4553*	*Camelliasinensis*	China	KX895012	KX895345	KX895231	Liu et al. (2017)
*Pes.yunnanensis*	HMAS 96359*	*Podocarpusmacrophyllus*	China	AY373375	NA	NA	Wei et al. (2013)
*Pes.zhaoqingensis*	ZHKUCC 23-0825*	dead leaves of unknown host	China	OR233336	OR239062	OR239061	Dong et al. (2023)
*Neopestalotiopsismagna*	MFLUCC 12-0652*	*Pteridium* sp.	France	KF582795	KF582793	KF582791	Maharachchikumbura et al. (2014)

### ﻿Morphology

Morphological characteristics of the isolates were assessed using sporulating pure cultures grown on PDA and MEA under dark conditions at 25°C. The conidiomata were observed and photographed using a dissecting microscope (Carl Zeiss, Munich, Germany). The conidiogenous cells and conidia were mounted in one drop of sterile water on glass slides and examined under an Axio Imager A2 microscope (Carl Zeiss, Munich, Germany) and an AxioCam ERc 5S digital camera with Zeiss Axio Vision 4.8 software (Carl Zeiss) using differential interference contrast (DIC) illumination. Fifty measurements were recorded for each morphological structure of the holotype culture, while thirty measurements were taken for other cultures. Minimum, maximum and average (mean) values were determined and they are presented as follows: (minimum –) (average – standard deviation) – (average + standard deviation) (– maximum). To investigate the impact of temperature on mycelial growth of the identified *Pestalotiopsis* species, 5 mm diameter agar plugs containing mycelium were transferred from these cultures to fresh 2% MEA Petri dishes and incubated in the dark at 5°C to 35°C intervals at a temperature range of 5°C. Each experiment was replicated five times for each temperature. Once one strain completely covered the entire dish, colony diameter was measured and growth rate was calculated using orthogonal measurements. The isolated strains were subsequently inoculated on to fresh MEA, cultured at 25°C for seven days and colony characteristics were assessed. To facilitate comparison of species growing on pine hosts, the available measurement data are summarized in Table 3.

**Table 3.** Synopsis of *Pestalotiopsis* occurring on *Pinus* hosts.

Species	Length of conidia (μm)	Width of conidia (μm)	Length of 3 median cells (μm)	Length of apical appendage (μm)	Length of basal appendage (μm)	Hosts	Country	Reference
*Pes.clavata*	17–22.5	6–8.5	12–14	8.5–25.5	3–7	*Pinuselliottii*, *Pinusmassoniana*, *Pinusthunbergii*	China	This study
*Pes.disseminata*	8–26	4–8	13–16.5	3.5–22	3–5.5	*Pinuselliottii*, *Pinusthunbergii*	China	This study
*Pes.guangxiensis*	16–22	4–9.5	11–14.5	7–18.5	2.5–4.5	*Pinusmassoniana*	China	This study
*Pes.iberica*	16.1–30.7	4.5–7.3	9.8–19.3	3.8–16.0	1.4–10.2	*Pinusradiata*, *Pinussylvestris*	Spain	Monteiro et al. (2022)
*Pes.jiangmenensis*	18–25	5–7.5	11–16	5.5–24	2.5–8.5	*Pinusmassoniana*	China	This study
*Pes.jiangsuensis*	20.3–27.3	6.2–8.7	12.7–16.56	8.7–23.4	1.4–6.3	*Pinusmassoniana*	China	Li et al. (2024)
*Pes.lushanensis*	18–25	6–8	10.5–16	7.5–22.5	3.5–5.5	*Pinusmassoniana*	China	This study
*Pes.massoniana*	17–21	5–7	10.5–16	2.7–18	1.5–4.5	*Pinusmassoniana*	China	This study
*Pes.neglecta*	19.8–25.5	5.8–7.5	NA	18.8–28.0	NA	*Pinussylvestris*	China	Chen et al. (2020)
*Pes.ningboensis*	21–27	6–8	12.5–17	5.5–19	2–9	*Pinuselliottii*	China	This study
*Pes.pini*	20.0–27.6	4.7–8.2	12.2–17.3	9.7–27.8	1.4–7.6	*Pinuspinaster*, *Pinuspinea*	Portugal	Silva et al. (2020)
*Pes.pinicola*	18–23	5–7	11.0–16.0	5–17	2.0–7.0	*Pinusarmandii*	China	Tibpromma et al. (2019)
*Pes.rosea*	17.5–21.8	5.7–7	11.8–13.8	14–22	2–5.7	*Pinus* sp.	China	Maharachchikumbura et al. (2012)
*Pes.shanweiensis*	17.5–22.5	5.5–7.5	11.5–14.5	6–22	3–6.5	*Pinusmassoniana*	China	This study
*Pes.thunbergii*	20–34	6–10	13.5–19	6.5–29.5	3.5–8	*Pinusmassoniana*, *Pinusthunbergii*	China	This study
*Pes.trachicarpicola*	21.8–28.8	9 5.5–8.5	NA	9.8–25.3	3.6–8.2	*Pinusbungeana*	China	Qi et al. (2021)
*Pes.wenzhouensis*	20–27.5	7–8	12.5–17.5	7–19.5	2.5–6	*Pinusmassoniana*	China	This study

## ﻿Results

### ﻿Fungal isolation

In total, 209 fresh samples of diseased pine needles with blight symptoms were collected from nine sampling sites in Zhejiang, Shandong and Guangdong Provinces in China, representing different climate zones. A total of 309 strains were isolated, 100 of which were identified as the genus of *Pestalotiopsis*. The representative isolates from each sample were selected, leading to a number of 74 strains for further study.

### ﻿Phylogenetic analyses

**Figure 3.**
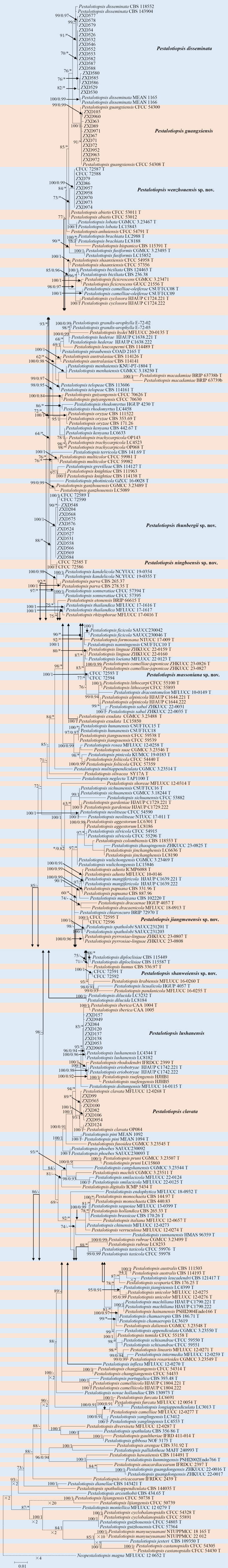
Phylogenetic trees based on Maximum Likelihood (ML) analyses from the multi-gene alignment (ITS, *tef-1α* and *tub2*) for *Pestalotiopsis*. The ML bootstrap values (left; values of ≥ 60% are shown, < 60% are marked with * and absence is marked with -) and Bayesian posterior probabilities (right; values of ≥ 0.9 are shown, < 0.9 are marked with * and absence is marked with -) are indicated above the branches. Isolates representing ex-type material are marked with “T”. *Neopestalotiopsismagna* (MFLUCC 12-0652) was used as the outgroup taxon.

The combined DNA sequence datasets of ITS, *tef1-α* and *tub2* were aligned and used to infer delimitation for *Pestalotiopsis*. The alignment comprises 1936 characters including alignment gaps after alignment (597 for ITS, 736 for *tef1-α* and 603 for *tub2*) and 620 were parsimony informative. The topology of phylogenetic trees constructed from the combined sequence data of ITS, *tef1-α* and *tub2* remained generally consistent, although the relative position of some *Pestalotiopsis* species was slightly different between the ML and BI trees. The ML trees are shown. Isolates from the present study formed ten individual clades representing ten species of *Pestalotiopsis*, including six undescribed species, which are described here: *Pes.jiangmenensis*, *Pes.massoniana*, *Pes.ningboensis*, *Pes.shanweiensis*, *Pes.thunbergii* and *Pes.wenzhouensis* and four known species (*Pes.clavata*, *Pes.disseminata*, *Pes.guangxiensis* and *Pes.lushanensis*) (Fig. 3).

### ﻿Taxonomy

#### ﻿ Pestalotiopsis clavata

Taxon classificationAnimaliaAmphisphaerialesSporocadaceae

Maharachch. & K.D. Hyde, Fungal. Divers. 56: 108 (2012).

C0CD2937-B4FC-5832-8F6D-E35B3F27FA01

Fig. 4

##### Description.

Sexual state not seen. ***Conidiomata*** in culture sporodochial, saucer-shaped, scattered or gregarious, superficial to immersed, shining, releasing black conidial masses on the surface. ***Conidiophores*** branched, subcylindrical, hyaline to light brown, indistinct, often reduced to conidiogenous cells. ***Conidiogenous cells*** cylindrical or ampulliform, hyaline, smooth-walled, solitary to aggregated, (6–)7–9(–9.5) × (2–)2.5–3.5 μm (x ± SD = 7.8 ± 1 × 2.8 ± 0.5 μm). ***Conidia*** fusoid, ellipsoid, smooth, slightly constricted at the septa, 4-septate, (17–)19.5–21.5(–22.5) × (6–)6.5–8(–8.5) μm (x ± SD = 20.5 ± 1.2 × 7.4 ± 0.7 μm); apical cell with 2–3 tubular appendages (mostly three); apical appendages arising from an apical crest, unbranched, filiform, bent, (8.5–)11–19(–25.5) μm (x ± SD = 15 ± 3.8 μm), basal cell with one appendage; basal appendage tubular, centric, unbranched, occasionally swollen at the tip, (3–)4–6.5(–7) μm (x ± SD = 5.2 ± 1.2 μm) long.

##### Materials examined.

CHINA • Zhejiang Province, Wenzhou City, Cangnan County, Dayu Town, 27°22'37"N, 120°36'48"E, on diseased needle of *Pinusmassoniana*, 6 May 2023, *Quanchao Wang, Guiyong Cao* (cultures ZXD82, ZXD99, ZXD100, ZXD106); • Zhejiang Province, Wenzhou City, Cangnan County, Zaoxi Town, 27°26'6"N, 120°29'40"E, on diseased needle of *Pinusmassoniana*, 6 May 2023, *Quanchao Wang, Guiyong Cao* (culture ZXD124); • Shandong Province, Qingdao City, Jimo District, Tianheng Island Resort, 36°25'2"N, 120°51'37"E, on diseased needle of *Pinusthunbergii*, 9 August 2023, *Quanchao Wang, Guoqing Li, Feifei Liu & Runlei Chang* (culture ZXD565); • Guangdong Province, Jiangmen City, Taishan County, Chixi Town, 21°53'10"N, 112°57'0"E, on diseased needle of *Pinusmassoniana*, 28 August 2023, *Quanchao Wang, Guoqing Li, Feifei Liu & Yuhua Liang* (culture ZXD954).

**Figure 4.**
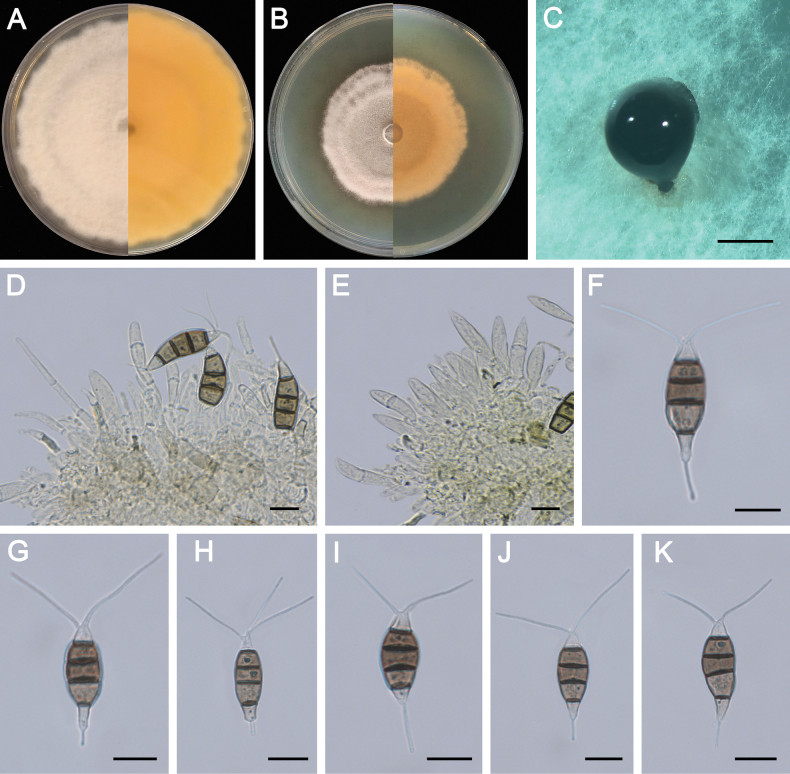
Morphology of *Pestalotiopsisclavata* (ZXD954). **A** Colony on MEA after 10 days at 25°C; **B** colony on PDA after 10 days at 25°C; **C** conidiomata formed on MEA; **D, E** conidiogenous cells giving rise to conidia; **F–K** conidia. Scale bars: 500 μm (**C**); 20 μm (**D–G**).

##### Notes.

*Pestalotiopsisclavata* was first reported from *Buxus* sp. in China (Maharachchikumbura et al. 2012). In this study, seven strains were isolated from *P.massoniana* and *P.thunbergii* and clustered together with *Pes.clavata* in the multi-locus phylogenetic tree (Fig. 3). Compare with the description of ex-type isolate MFLUCC 12-0268, ZXD954 has shorter appendages (3–7 μm vs. 7–9 μm), while other morphological characteristics are similar. This is the first report of *Pes.clavata* on the host of *P.massoniana* and *P.thunbergii*.

#### ﻿ Pestalotiopsis disseminata

Taxon classificationAnimaliaAmphisphaerialesSporocadaceae

(Thüm.) Steyaert, Bull. Jard. bot. Etat Brux. 19: 319. (1949).

200DE849-197C-5839-A042-80CAFC9B0A1D

Fig. 5

##### Description.

Sexual state not seen. ***Conidiomata*** in culture sporodochial, saucer-shaped, scattered or gregarious, superficial to immersed, shining, releasing black conidial masses on the surface. ***Conidiophores*** branched, subcylindrical, hyaline to light brown, indistinct, often reduced to conidiogenous cells. ***Conidiogenous cells*** cylindrical or ampulliform, hyaline, smooth-walled, solitary to aggregated, (5.5–)7–10(–11.5) × 2–3 μm (x ± SD = 8.4 ± 1.6 × 2.4 ± 0.4 μm). ***Conidia*** fusoid, ellipsoid, smooth, slightly constricted at the septa, 4-septate, (8–)17.5–25.5(–26) × (4–)6–7.5(–8) μm (x ± SD = 21.6 ± 4.1 × 6.7 ± 0.8 μm); apical cell with 2–3 tubular appendages (mostly three); apical appendages arising from an apical crest, unbranched, filiform, bent, (3.5–)10.5–16.5(–22) μm (x ± SD = 13.6 ± 3.1 μm) long; basal cell with one appendage; basal appendage tubular, centric appendage tubular, unbranched, occasionally swollen at the tip, (2–)2.5–4(–4.5) μm (x ± SD = 3.4 ± 0.7 μm) long.

**Figure 5.**
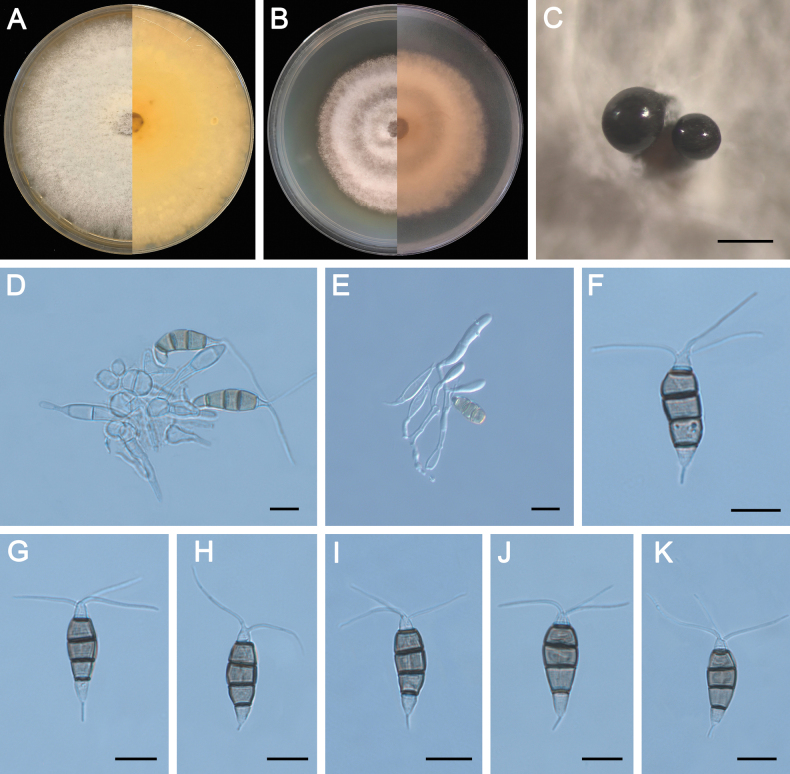
Morphology of *Pestalotiopsisdisseminata* (ZXD579). **A** Colony on MEA after 10 days at 25°C; **B** colony on PDA after 10 days at 25°C; **C** conidiomata formed on MEA; **D, E** conidiogenous cells giving rise to conidia; **F–K** conidia. Scale bars: 500 μm (**C**); 20 μm (**D–G**).

##### Materials examined.

CHINA • Zhejiang Province, Ningbo City, Xiangshan County, Fengmenkou Forest Park, 29°5'44"N, 121°57'19"E, on diseased needle of *Pinuselliottii*, 19 April 2023, *Quanchao Wang & Guiyong Cao* (culture ZXD34); • Shandong Province, Qingdao City, Jimo District, Tianheng Island Resort, 36°25'2"N, 120°51'37"E, on diseased needle of *Pinusthunbergii*, 9 August 2023, *Quanchao Wang, Guoqing Li, Feifei Liu & Runlei Chang* (cultures ZXD526, ZXD529, ZXD530, ZXD532, ZXD546, ZXD552, ZXD553, ZXD577, ZXD578, ZXD579, ZXD580, ZXD582, ZXD585, ZXD586, ZXD587, ZXD588).

##### Notes.

*Pestalotiopsisdisseminata* was first reported from *Eucalyptusbotryoides* in Portugal (Von Thümen 1881) and subsequently reported on a wide range of hosts and locations, including the genus *Pinus* (Hu et al. 2007; Liu et al. 2019; Silva et al. 2020). In this study, 17 strains were isolated from *P.elliottii* and *P.thunbergii* and clustered together with *Pes.disseminata* in the multi-locus phylogenetic tree (Fig. 3). Compared to the description of the isolate CBS 118552, ZXD579 exhibits smaller conidiogenous cells (5.5–11.5 × 2–3 μm vs. 7–24.5 × 2–5 μm), while other morphological characteristics are similar. This is the first report of *Pes.disseminata* on the host of *P.elliottii* and *P.thunbergii*.

#### ﻿ Pestalotiopsis guangxiensis

Taxon classificationAnimaliaAmphisphaerialesSporocadaceae

Ning Jiang, Microbiol. Spectr. 6: 14 (2022).

1FE8C416-8CAE-54D8-A910-90FB865E54C5

Fig. 6

##### Description.

Sexual state not seen. ***Conidiomata*** in culture sporodochial, saucer-shaped, scattered or gregarious, superficial to immersed, shining, releasing black conidial masses on the surface. ***Conidiophores*** branched, subcylindrical, hyaline to light brown, indistinct, often reduced to conidiogenous cells. ***Conidiogenous cells*** cylindrical or ampulliform, hyaline, smooth-walled, solitary to aggregated, (7.5–)9–13(–14.5) × (1.5–)3–4(–5) μm (x ± SD = 11 ± 1.9 × 3.5 ± 0.7 μm). ***Conidia*** fusoid, ellipsoid, smooth, slightly constricted at the septa, 4-septate, (16–)17.5–20.5(–22) × (4–)6.5–9(–9.5) μm (x ± SD = 19 ± 1.6 × 7.6 ± 1.2 μm); apical cell conic with 2–4 tubular appendages (mostly four); apical appendages arising from an apical crest, unbranched, filiform, bent, (7–)11–16(–18.5) μm (x ± SD = 13.4 ± 3.3 μm); basal cell obconic with one appendage, basal appendage tubular, centric appendage tubular, unbranched, occasionally swollen at the tip, (2.5–)3–4(–4.5) μm (x ± SD = 3.4 ± 0.6 μm) long.

##### Materials examined.

CHINA • Zhejiang Province, Wenzhou City, Cangnan County, Dayu Town, 27°22'37"N, 120°36'48"E, on diseased needle of *Pinusmassoniana*, 6 May 2023, *Quanchao Wang, Guiyong Cao* (cultures ZXD63, ZXD67, ZXD71, ZXD72, ZXD89, ZXD103); • Guangdong Province, Shanwei City, Zhelang Street, 22°47'29"N, 115°32'18"E, on diseased leaves of *Pinusmassoniana*, 27 August 2023, *Quanchao Wang, Guoqing Li, Feifei Liu & Runlei Chang* (culture ZXD952); • Guangdong Province, Jiangmen City, Taishan County, Chixi Town, 21°53'10"N, 112°57'0"E, on diseased needle of *Pinusmassoniana*, 28 August 2023, *Quanchao Wang, Guoqing Li, Feifei Liu & Yuhua Liang* (cultures ZXD960, ZXD963, ZXD971, ZXD972).

**Figure 6.**
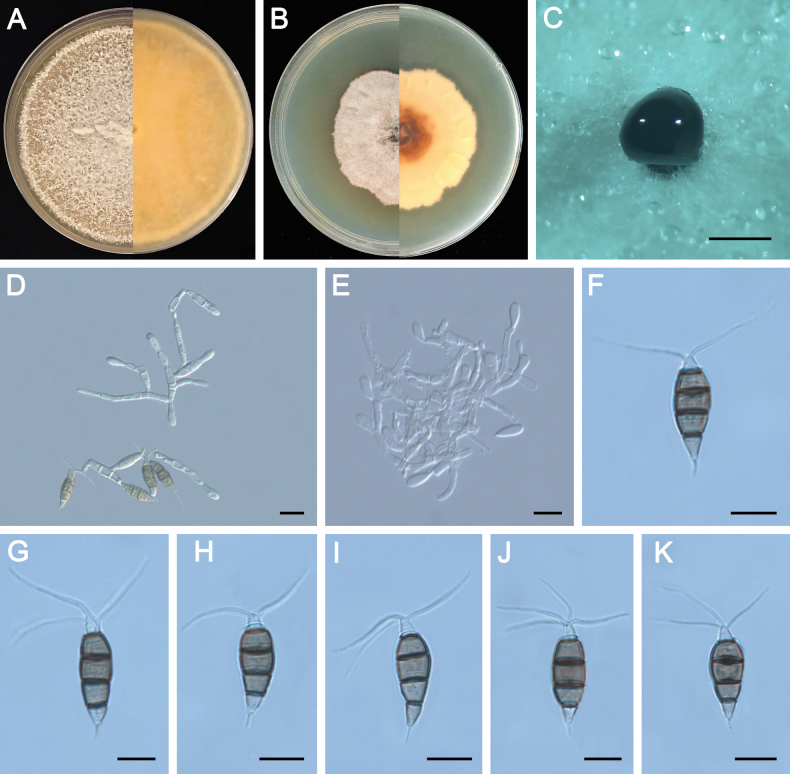
Morphology of *Pestalotiopsisguangxiensis* (ZXD89). **A** Colony on MEA after 10 days at 25°C; **B** colony on PDA after 10 days at 25°C; **C** conidiomata formed on MEA; **D, E** conidiogenous cells giving rise to conidia; **F–K** conidia. Scale bars: 500 μm (**C**); 20 μm (**D–G**).

##### Notes.

*Pestalotiopsisguangxiensis* was first reported from *Quercusgriffithii* in China (Jiang et al. 2022c). In this study, 11 strains were isolated from *P.massoniana* and clustered together with *Pes.guangxiensis* in the multi-locus phylogenetic tree (Fig. 3). Compared with the description of ex-type isolate CFCC 54308 (Jiang et al. 2022c), isolate ZXD960 has shorter apical appendages (7–18.5 μm vs. 14–19 μm), while other morphological characteristics are similar. This is the first report of *Pes.guangxiensis* on the host of *P.massoniana*.

#### ﻿ Pestalotiopsis jiangmenensis

Taxon classificationAnimaliaAmphisphaerialesSporocadaceae

Q.C. Wang & X.D. Zhou sp. nov.

0226F1E4-160E-5125-A052-7E7B6171CFF5

MB858311

Fig. 7

##### Etymology.

Named after the collection site of the type specimen, Jiangmen City.

##### Typus.

CHINA • Guangdong Province, Jiangmen City, Taishan County, Chixi Town, 21°53'10"N, 112°57'0"E, on diseased needle of *Pinusmassoniana*, 28 August 2023, *Quanchao Wang, Guoqing Li, Feifei Liu & Yuhua Liang* (holotype designated here HMAS 353944, dried culture prepared from ZXD964; ex-holotype culture ZXD964 = CFCC 72595).

##### Description.

Sexual state not seen. ***Conidiomata*** in culture sporodochial, saucer-shaped, scattered or gregarious, superficial to immersed, shining, releasing black conidial masses on the surface. ***Conidiophores*** branched, subcylindrical, hyaline to light brown, indistinct, often reduced to conidiogenous cells. ***Conidiogenous cells*** cylindrical or ampulliform, hyaline, smooth-walled, solitary to aggregated, (2.5–)6–11.5(–14) × (0.5–)1.5–3(–3.5) μm (x ± SD = 8.9 ± 2.7 × 2.4 ± 0.7 μm). ***Conidia*** fusoid, ellipsoid, smooth, slightly constricted at the septa, 4-septate, (18–)19.5–22.5(–25) × (5–)5.5–7(–7.5) μm (x ± SD = 20.9 ± 1.5 × 6.2 ± 0.6 μm); three median cells doliiform, wall verruculose, concolourous, (11–)12–14.5(–16) μm (x ± SD = 13.3 ± 1.2 μm) long; second cell from the base (3.5–)4–5(–5.5) μm (x ± SD = 4.4 ± 0.5 μm) long; third cell (3.5–)4–5(–6) μm (x ± SD = 4.4 ± 0.5 μm); fourth cell (3.5–)4–5(–6) μm (x ± SD = 4.5 ± 0.6 μm); apical cell conic with an acute apex, thin- and smooth-walled, hyaline to pale brown, (2.5–)3–4(–4.5) μm (x ± SD = 3.5 ± 0.6 μm) long, with 2–4 tubular appendages (mostly three); apical appendages arising from an apical crest, unbranched, filiform, bent, (5.5–)10–15.5(–24) μm (x ± SD = 12.7 ± 2.7 μm); basal cell obconic with a truncate base, thin-walled, hyaline or pale brown, (3–)3.5–4.5(–5) μm (x ± SD = 4.1 ± 0.5 μm) long, with one appendage, tubular; basal appendage centric, tubular, unbranched, occasionally swollen at the tip, (2.5–)4–6(–8.5) μm (x ± SD = 5 ± 1.2 μm) long.

**Figure 7.**
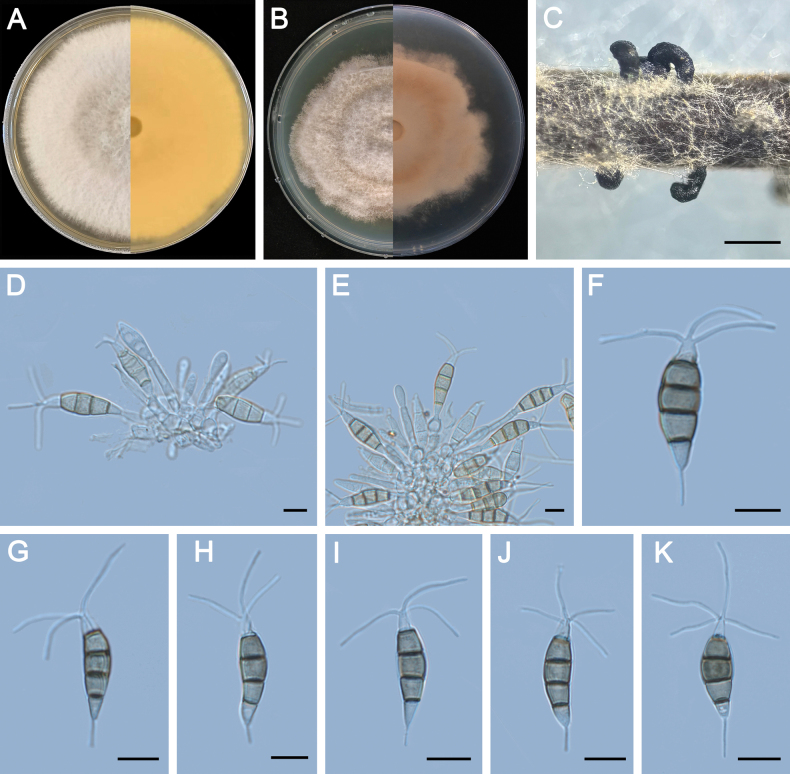
Morphology of *Pestalotiopsisjiangmenensis* (ZXD964). **A** Colony on MEA after 10 days at 25°C; **B** colony on PDA after 10 days at 25°C; **C** conidiomata formed on MEA; **D, E** conidiogenous cells giving rise to conidia; **F–K** conidia. Scale bars: 500 μm (**C**); 20 μm (**D–G**).

##### Culture characteristics.

**Figure 8.**
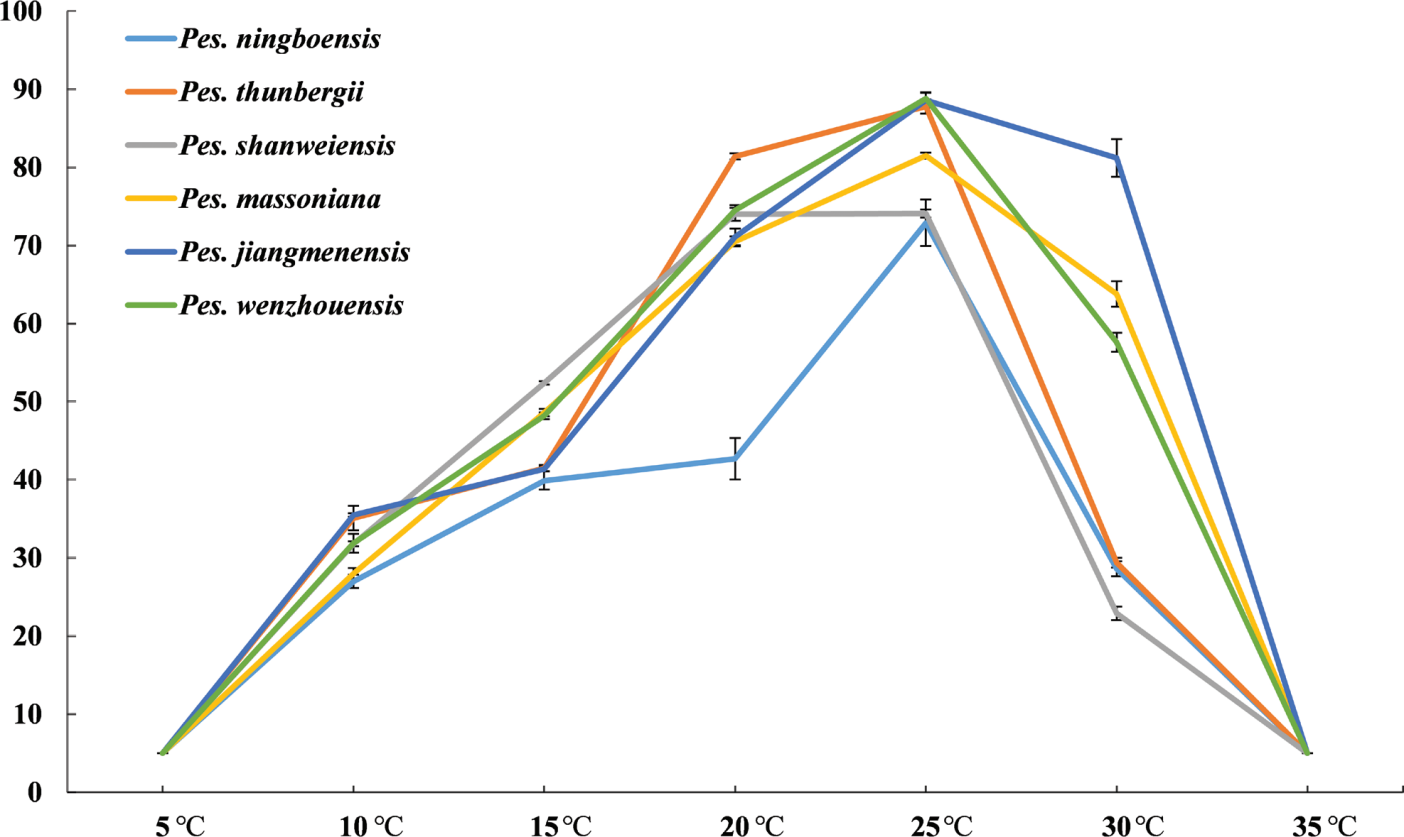
Effect of temperature on mycelial radial growth of isolates of six new *Pestalotiopsis* species obtained in the current study. Vertical bars represent the standard error of the means. Each value represents the average of five replicates.

Colonies exhibited abundant flocculent aerial mycelium on PDA at 25°C, with irregular margins at the edges, while the reverse side of the colony appeared light orange, forming black conidiomata with black conidial masses. Optimal growth temperature at 25°C, no growth at 5°C and 35°C; after 10 d, colonies at 10, 15, 20, 25 and 30°C reached 35.5, 41.4, 71.1, 88.6 and 81.2 mm, respectively (Fig. 8).

##### Additional materials examined.

CHINA • Guangdong Province, Jiangmen City, Taishan County, Chixi Town, 21°53'10"N, 112°57'0"E, on diseased needle of *Pinusmassoniana*, 28 August 2023, *Quanchao Wang, Guoqing Li, Feifei Liu & Yuhua Liang* (culture ZXD965).

##### Notes.

*Pestalotiopsisjiangmenensis* forms a well-supported independent clade (ML/BI = 100/1) and is phylogenetically distinct from *Pes.pyrrosiae-linguae* and *Pes.spatholobi* (Fig. 3). In detail, *Pes.jiangmenensis* was distinguished from *Pes.pyrrosiae-linguae* and *Pes.spatholobi* in ITS (*Pes.pyrrosiae-linguae* 4 bp, *Pes.spatholobi*: 4 bp); *tef1-α* gene (*Pes.pyrrosiae-linguae*: 1 bp, *Pes.spatholobi*: 1 bp); *tub2* gene (*Pes.pyrrosiae-linguae*: 2 bp, *Pes.spatholobi*: 2 bp). Morphologically, *Pes.jiangmenensis* can be distinguished from *Pes.pyrrosiae-linguae* and *Pes.spatholobi* by its shorter, but more numerous apical appendages (*Pes.jiangmenensis*: 5.5–24 μm, n = 2–4; *Pes.pyrrosiae-linguae*: 9–25 μm, n = 2–3; *Pes.spatholobi*: 8.4–15.3 μm, n = 1–3). Notably, *Pes.jiangmenensis* exhibits shorter basal appendages than *Pes.pyrrosiae-linguae*, but longer ones than *Pes.spatholobi* (*Pes.jiangmenensis*: 2.5–8.5 μm; *Pes.pyrrosiae-linguae*: 4.5–13 μm; *Pes.spatholobi*: 0.9–3.1 μm). Based on both phylogenetic and morphological evidence, we propose the recognition of *Pes.jiangmenensis* as a novel species.

#### ﻿ Pestalotiopsis lushanensis

Taxon classificationAnimaliaAmphisphaerialesSporocadaceae

F. Liu & L. Cai, Sci. Rep. 7: 9 (2017).

ED0EAEAE-8559-5CE9-945C-354F83391E1D

Fig. 9

##### Description.

Sexual state not seen. ***Conidiomata*** in culture sporodochial, saucer-shaped, scattered or gregarious, superficial to immersed, shining, releasing black conidial masses on the surface. ***Conidiophores*** branched, subcylindrical, hyaline to light brown, indistinct, often reduced to conidiogenous cells. ***Conidiogenous cells*** cylindrical or ampulliform, hyaline, smooth-walled, solitary to aggregated, (6.5–)9–14.5(–17.5) × (2–)2.5–3.5(–4) μm (x ± SD = 11.7 ± 2.6 × 3 ± 0.5 μm). ***Conidia*** fusoid, ellipsoid, smooth, slightly constricted at the septa, 4-septate, (18–)20–24(–25) × (6–)6.5–8 μm (x ± SD = 21.8 ± 2 × 7.3 ± 0.6 μm); apical cell conic with 2–4 tubular apical appendages (mostly four), arising from an apical crest, unbranched, filiform, bent, (7.5–)11–17.5(–22.5) μm (x ± SD = 14.1 ± 3.2 μm); basal cell obconic with one basal appendage, tubular, centric appendage tubular, unbranched, occasionally swollen at the tip, 3.5–5(–5.5) μm (x ± SD = 4.3 ± 0.6 μm) long.

**Figure 9.**
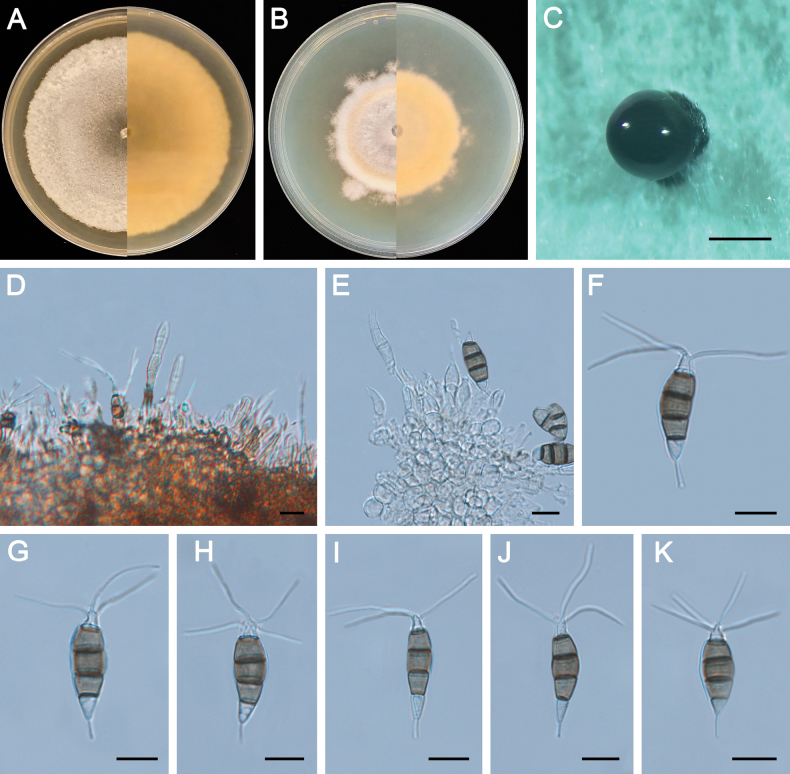
Morphology of *Pestalotiopsislushanensis* (ZXD969). **A** Colony on MEA after 10 days at 25°C; **B** colony on PDA after 10 days at 25°C; **C** conidiomata formed on MEA; **D, E** conidiogenous cells giving rise to conidia; **F–K** conidia. Scale bars: 500 μm (**C**); 20 μm (**D–G**).

##### Materials examined.

CHINA • Zhejiang Province, Wenzhou City, Cangnan County, Dayu Town, 27°22'37"N, 120°36'48"E, on diseased needle of *Pinusmassoniana*, 6 May 2023, *Quanchao Wang, Guiyong Cao* (culture ZXD84); • Zhejiang Province, Wenzhou City, Cangnan County, Zaoxi Town, 27°26'6"N, 120°29'40"E, on diseased needle of *Pinusmassoniana*, 6 May 2023, *Quanchao Wang, Guiyong Cao* (cultures ZXD120, ZXD137, ZXD138); • Zhejiang Province, Jiaxing City, Pinghu County, Jiulongshan Forest Park, 30°36'10"N, 121°08'27"E, on diseased needle of *Pinusmassoniana*, 15 May 2023, *Quanchao Wang, Guiyong Cao* (culture ZXD157); • Guangdong Province, Shanwei City, Zhelang Street, 22°47'29"N, 115°32'18"E, on diseased leaves of *Pinusmassoniana*, 27 August 2023, *Quanchao Wang, Guoqing Li, Feifei Liu & Runlei Chang* (culture ZXD949); • Guangdong Province, Jiangmen City, Taishan County, Chixi Town, 21°53'10"N, 112°57'0"E, on diseased needle of *Pinusmassoniana*, 28 August 2023, *Quanchao Wang, Guoqing Li, Feifei Liu & Yuhua Liang* (cultures ZXD953, ZXD969).

##### Notes.

*Pestalotiopsislushanensis* was first reported from *Camellia* sp. in China (Liu et al. 2017). In addition, this species was discovered on *Sarcandraglabra* and *Podocarpusmacrophyllus* (Zhang et al. 2021; Zheng et al. 2022). In this study, eight strains were isolated from *P.massoniana* and clustered together with *Pes.lushanensis* in the multi-locus phylogenetic tree (Fig. 3). Compared with the description of ex-type isolate CGMCC 3.18160 (Liu et al. 2017), ZXD969 has shorter apical appendages (7.5–22.5 μm vs. 17–26 μm), while other morphological characteristics are similar. The *Pes.lushanensis* isolates identified in this study were exclusively isolated from *P.massoniana*. Notably, this fungal species has also been previously documented on various other pine hosts, including *P.armandii*, *P.elliottii*, *P.tabuliformis* and *P.yunnanensis* (Jiang et al. 2022a).

#### ﻿ Pestalotiopsis massoniana

Taxon classificationAnimaliaAmphisphaerialesSporocadaceae

Q.C. Wang & X.D. Zhou sp. nov.

8BC2BD89-E9C4-511B-BDDB-0ECE22D8643E

MB858312

Fig. 10

##### Etymology.

Named after the host, *Pinusmassoniana*.

##### Typus.

CHINA • Guangdong Province, Jiangmen City, Taishan County, Chixi Town, 21°53'10"N, 112°57'0"E, on diseased needle of *Pinusmassoniana*, 28 August 2023, *Quanchao Wang, Guoqing Li, Feifei Liu & Yuhua Liang* (holotype designated here HMAS 353942, dried culture prepared from ZXD955; ex-holotype culture ZXD955 = CFCC 72593).

##### Description.

Sexual state not seen. ***Conidiomata*** in culture sporodochial, saucer-shaped, scattered or gregarious, superficial to immersed, shining, releasing black conidial masses on the surface. ***Conidiophores*** branched, subcylindrical, hyaline to light brown, indistinct, often reduced to conidiogenous cells. ***Conidiogenous cells*** cylindrical or ampulliform, hyaline, smooth-walled, solitary to aggregated, (6–)7–10.5(–13) × (2–)2.5–3.5(–4) μm (x ± SD = 8.9 ± 1.8 × 3.1 ± 0.5 μm). ***Conidia*** fusoid, ellipsoid, smooth, slightly constricted at the septa, 4-septate, (17–)18–20(–21) × (5–)5.5–6.5(–7) μm (x ± SD = 19.1 ± 1.1 × 6.2 ± 0.5 μm); three median cells doliiform, wall verruculose, concolourous, (10.5–)12–14(–16) μm (x ± SD = 12.9 ± 1.1 μm) long; second cell from the base (3–)4–5 μm (x ± SD = 4.4 ± 0.4 μm) long; third cell (3–)3.5–4.5(–5) μm (x ± SD = 4 ± 0.4 μm); fourth cell (3–)4–5(–7) μm (x ± SD = 4.5 ± 0.7 μm); apical cell conic with an acute apex, thin- and smooth-walled, hyaline to pale brown, (2–)2.5–4(–4.5) μm (x ± SD = 3.3 ± 0.6 μm) long, with 2–3 tubular appendages (mostly three); apical appendages arising from an apical crest, unbranched, filiform, bent, (2.7–)6.5–13(–18) μm (x ± SD = 9.8 ± 3.3 μm); basal cell obconic with a truncate base, thin-walled, hyaline or pale brown, 2.5–4(–6.5) μm (x ± SD = 3.4 ± 0.8 μm) long, with one appendage; basal appendage tubular, centric appendage tubular, unbranched, occasionally swollen at the tip, (1.5–)2–4(–4.5) μm (x ± SD = 2.9 ± 0.9 μm) long.

**Figure 10.**
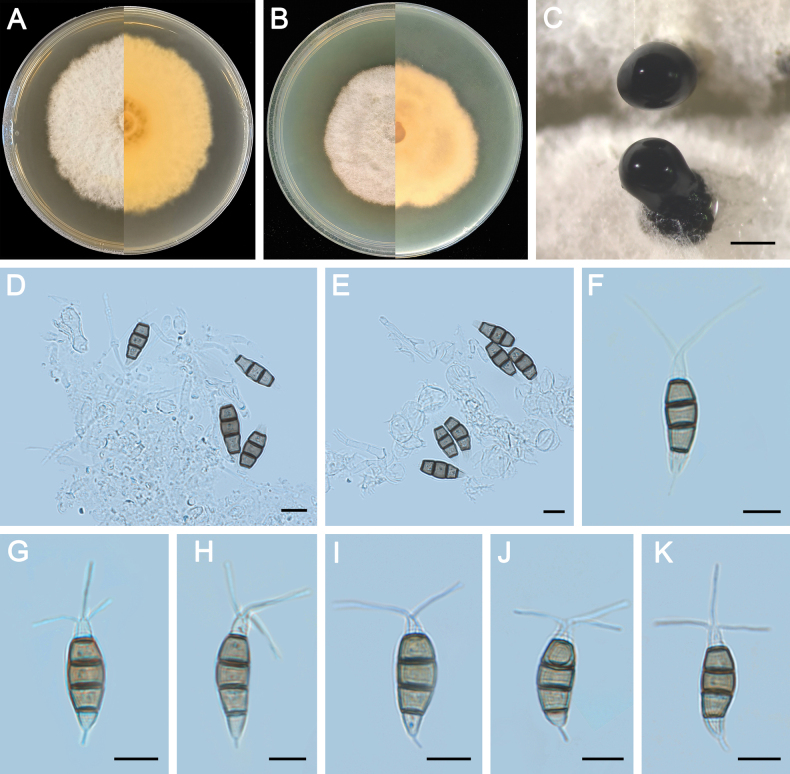
Morphology of *Pestalotiopsismassoniana* (ZXD955). **A** Colony on MEA after 10 days at 25°C; **B** colony on PDA after 10 days at 25°C; **C** conidiomata formed on MEA; **D, E** conidiogenous cells giving rise to conidia; **F–K** conidia. Scale bars: 500 μm (**C**); 20 μm (**D–G**).

##### Culture characteristics.

Colonies forming abundant flocculent aerial mycelium on PDA at 25°C, with regular margins at the edges, with a white appearance, while the reverse side of the colony displayed a light orange colour and forming black conidiomata with black conidial masses. Optimal growth temperature at 25°C, no growth at 5°C and 35°C; after 10 d, colonies at 10, 15, 20, 25 and 30°C reached 28.0, 48.6, 70.5, 81.5 and 63.8 mm, respectively (Fig. 8).

##### Additional materials examined.

CHINA • Guangdong Province, Jiangmen City, Taishan County, Chixi Town, 21°53'10"N, 112°57'0"E, on diseased needle of *P.massoniana*, 28 August 2023, *Quanchao Wang, Guoqing Li, Feifei Liu & Yuhua Liang* (culture ZXD956).

##### Notes.

*Pestalotiopsismassoniana* forms a well-supported independent clade (ML/BI = 100/1) and is phylogenetically distinct from *Pes.alpinicola*, *Pes.dracontomelon*, *Pes.lithocarpi* and *Pes.sabal*. (Fig. 3). In detail, *Pes.massoniana* was distinguished from *Pes.alpinicola*, *Pes.dracontomelon*, *Pes.lithocarpi* and *Pes.sabal* in ITS (*Pes.alpinicola*: 3 bp, *Pes.dracontomelon*: 3 bp, *Pes.lithocarpi*: 4 bp, *Pes.sabal*: 4 bp); *tef1-α* gene (*Pes.alpinicola*: 22 bp, *Pes.dracontomelon*: 30 bp, *Pes.lithocarpi*: 27 bp, *Pes.sabal*: 24 bp); *tub2* gene (*Pes.alpinicola*: 0 bp, *Pes.dracontomelon*: no data, *Pes.lithocarpi*: 1 bp, *Pes.sabal*: 2 bp). Morphologically, *Pes.massoniana* shares comparable conidial dimensions with congeners, but exhibits diagnostically shorter apical appendages from *Pes.dracontomelon* and *Pes.lithocarpi* (*Pes.massoniana*: 10.5–16 μm; *Pes.dracontomelon*: 13–17 μm; *Pes.lithocarpi*: 12.5–14.5 μm). Similarly, *Pes.massoniana* possesses significantly shorter basal appendages compared to *Pes.alpinicola*, *Pes.dracontomelon* and *Pes.sabal* (*Pes.massoniana*: 1.5–4.5 μm; *Pes.alpinicola*: 3.6–6.2 μm, *Pes.dracontomelon*: 2–7 μm, *Pes.sabal*: 3–5.5 μm). Based on both phylogenetic and morphological evidence, we propose the recognition of *Pes.massoniana* as a novel species.

#### ﻿ Pestalotiopsis ningboensis

Taxon classificationAnimaliaAmphisphaerialesSporocadaceae

Q.C. Wang & X.D. Zhou sp. nov.

6CD00197-BFB1-50E5-9B64-539A338CD9BB

MB858313

Fig. 11

##### Etymology.

Named after the collection site of the type specimen, Ningbo City.

##### Typus.

CHINA • Zhejiang Province, Ningbo City, Xiangshan County, Fengmenkou Forest Park, 29°5'44"N, 121°57'19"E, on diseased needle of *Pinuselliottii*, 19 April 2023, *Quanchao Wang & Guiyong Cao* (holotype designated here HMAS 353934, dried culture prepared from ZXD39; ex-holotype culture ZXD39 = CFCC 72585).

##### Description.

Sexual state not seen. ***Conidiomata*** in culture sporodochial, saucer-shaped, scattered or gregarious, superficial to immersed, shining, releasing black conidial masses on the surface. ***Conidiophores*** branched, subcylindrical, hyaline to light brown, indistinct, often reduced to conidiogenous cells. ***Conidiogenous*** cells cylindrical or ampulliform, hyaline, smooth-walled, solitary to aggregated, (5.5–)6.5–12.5(–17.5) × (1.5–)2.5–3.5(–4) μm (x ± SD = 9.5 ± 2.9 × 2.9 ± 0.6 μm). ***Conidia*** fusoid, ellipsoid, smooth, slightly constricted at the septa, 4-septate, (21–)21.5–25(–27) × (6–)6.5–7.5(–8) μm (x ± SD = 23.2 ± 1.8 × 6.8 ± 0.5 μm); three median cells doliiform, wall verruculose, concolourous, (12.5–)14–16(–17) μm (x ± SD = 15 ± 1 μm) long; second cell from the base (4–)4.5–5.5 μm (x ± SD = 4.9 ± 0.4 μm) long; third cell (4–)4.5–5.5 μm (x ± SD = 4.9 ± 0.4 μm); fourth cell (4–)4.5–5.5(–6) μm (x ± SD = 5.2 ± 0.5 μm); apical cell conic with an acute apex, thin- and smooth-walled, hyaline to pale brown, 3–4.5(–5.5) μm (x ± SD = 3.8 ± 0.6 μm) long, with 2–3 tubular appendages (mostly three); apical appendages arising from an apical crest, unbranched, filiform, bent, (5.5–)10.5–16.5(–19) μm (x ± SD = 13.5 ± 3 μm); basal cell obconic with a truncate base, thin-walled, hyaline or pale brown, (2–)3.5–5.5(–6) μm (x ± SD = 4.5 ± 1 μm) long, with one appendage; tubular; basal appendage centric, unbranched, occasionally swollen at the tip, (2–)5–8(–9) μm (x ± SD = 6.3 ± 1.5 μm) long.

**Figure 11.**
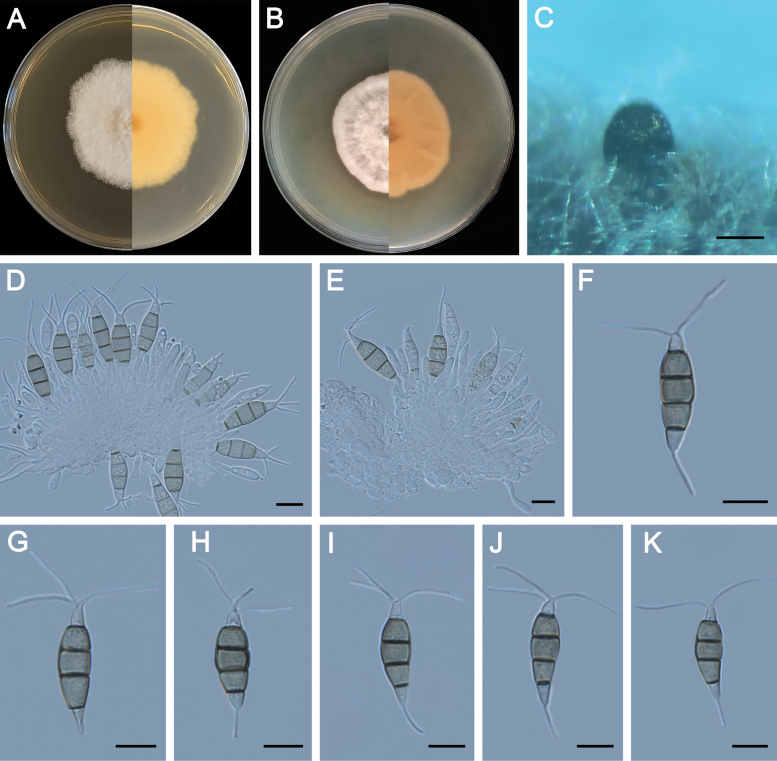
Morphology of *Pestalotiopsisningboensis* (ZXD40). **A** Colony on MEA after 10 days at 25°C; **B** colony on PDA after 10 days at 25°C; **C** conidiomata formed on MEA; **D, E** conidiogenous cells giving rise to conidia; **F–K** conidia. Scale bars: 500 μm (**C**); 20 μm (**D–G**).

##### Culture characteristics.

Colonies forming flocculent aerial mycelium on PDA at 25°C, with regular margins at the edges, white to isabelline, the back colony light orange, forming black conidiomata with black conidial masses. Optimal growth temperature at 25°C, no growth at 5°C and 35°C; after 10 d, colonies at 10, 15, 20, 25 and 30°C reached 27.0, 39.9, 42.7, 72.9 and 28.6 mm, respectively (Fig. 8).

##### Additional materials examined.

CHINA • Zhejiang Province, Ningbo City, Xiangshan County, Fengmenkou Forest Park, 29°5'44"N, 121°57'19"E, on diseased needle of *Pinuselliottii*, 19 April 2023, *Quanchao Wang & Guiyong Cao* (culture ZXD40).

##### Notes.

Two strains representing *Pes.ningboensis* form a well-supported independent clade (ML/BI = 100/1) and is phylogenetically distinct from *Pes.thunbergii* (Fig. 3), but differs in ITS (4 bp), *tub2* (6 bp) and *tef1-α* (4 bp). Morphologically, *Pes.ningboensis* can be distinguished from *Pes.thunbergii* by its shorter and narrower conidia (*Pes.ningboensis*: 21–27 × 6–8 μm vs. *Pes.thunbergii*: 20–34 × 6–10 μm); shorter apical appendages (*Pes.ningboensis*: 5.5–19 μm vs. *Pes.thunbergii*: 6.5–29.5 μm).

#### ﻿ Pestalotiopsis shanweiensis

Taxon classificationAnimaliaAmphisphaerialesSporocadaceae

Q.C. Wang & X.D. Zhou sp. nov.

11F9A590-551B-5898-9D5D-E02A545ECA12

MB858315

Fig. 12

##### Etymology.

Named after the collection site of the type specimen, Shanwei City.

##### Typus.

CHINA • Guangdong Province, Shanwei City, Zhelang Street, 22°47'29"N, 115°32'18"E, on diseased leaves of *Pinusmassoniana*, 27 August 2023, *Quanchao Wang, Guoqing Li, Feifei Liu & Runlei Chang* (holotype designated here HMAS 353940, dried culture prepared from ZXD950; ex-holotype culture ZXD950 = CFCC 72591).

##### Description.

Sexual state not seen. ***Conidiomata*** in culture sporodochial, saucer-shaped, scattered or gregarious, superficial to immersed, shining, releasing black conidial masses on the surface. ***Conidiophores*** branched, subcylindrical, hyaline to light brown, indistinct, often reduced to conidiogenous cells. ***Conidiogenous cells*** cylindrical or ampulliform, hyaline, smooth-walled, solitary to aggregated, (4–)5.5–10(–14) × 2–3.5(–5) μm (x ± SD = 7.7 ± 2.1 × 2.7 ± 0.7 μm). ***Conidia*** fusoid, ellipsoid, smooth, slightly constricted at the septa, 4-septate, (17.5–)19–21.5(–22.5) × (5.5–)6–7(–7.5) μm (x ± SD = 20.1 ± 1.2 × 6.7 ± 0.5 μm); three median cells doliiform, wall verruculose, concolourous; (11.5–)12–14(–14.5) μm (x ± SD = 13 ± 0.9 μm) long; second cell from the base (3.5–)4–5(–6) μm (x ± SD = 4.6 ± 0.5 μm) long; third cell (3–)4–5 μm (x ± SD = 4.3 ± 0.5 μm); fourth cell (3–)3.5–4.5 μm (x ± SD = 4.1 ± 0.5 μm); apical cell conic with an acute apex, thin- and smooth-walled, hyaline to pale brown, (2–)3–4 μm (x ± SD = 3.3 ± 0.5 μm) long, with 2–3 tubular appendages (mostly three); apical appendages arising from an apical crest, unbranched, filiform, bent, (6–)11–17(–22) μm (x ± SD = 14.1 ± 3 μm); basal cell obconic with a truncate base, thin-walled, hyaline or pale brown, (2.5–)3–4(–4.5) μm (x ± SD = 3.7 ± 0.6 μm) long, with one appendage; basal appendage tubular, centric, unbranched, occasionally swollen at the tip, (3–)4–5.5(–6.5) μm (x ± SD = 4.7 ± 0.9 μm) long.

**Figure 12.**
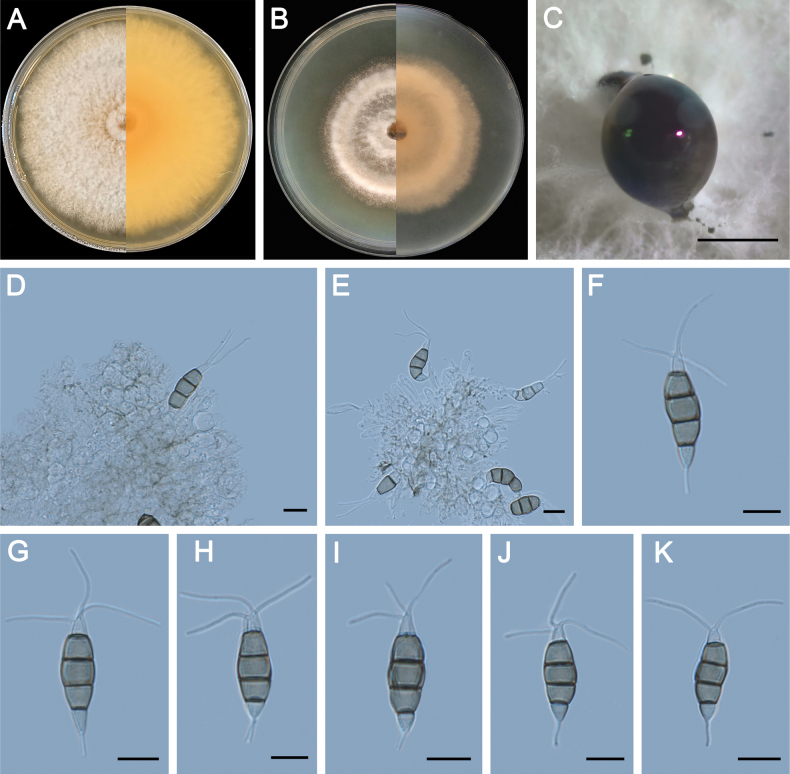
Morphology of *Pestalotiopsisshanweiensis* (ZXD951). **A** Colony on MEA after 10 days at 25°C; **B** colony on PDA after 10 days at 25°C; **C** conidiomata formed on MEA; **D, E** conidiogenous cells giving rise to conidia; **F–K** conidia. Scale bars: 500 μm (**C**); 20 μm (**D–G**).

##### Culture characteristics.

Colonies forming abundant flocculent aerial mycelium at the edges on PDA at 25°C, with regular margins, white to isabelline, the back colony light orange, forming black conidiomata with black conidial masses. Optimal growth temperature at 25°C, no growth at 5°C and 35°C; after 10 d, colonies at 10, 15, 20, 25 and 30°C reached 31.8, 52.4, 74, 74.1 and 22.9 mm, respectively (Fig. 8).

##### Additional materials examined.

CHINA • Guangdong Province, Shanwei City, Zhelang Street, 22°47'29"N, 115°32'18"E, on diseased leaves of *Pinusmassoniana*, 27 August 2023, *Quanchao Wang, Guoqing Li, Feifei Liu & Runlei Chang* (culture ZXD951).

##### Notes.

*Pestalotiopsisshanweiensis* forms a well-supported independent clade (ML/BI = 100/1) and is phylogenetically distinct from *Pes.licualicola*, *Pes.pandanicola* and *Pes.krabiensis* (Fig. 3). In detail, *Pes.shanweiensis* was distinguished from *Pes.licualicola*, *Pes.pandanicola* and *Pes.krabiensis* in ITS (*Pes.krabiensis*: 6 bp, *Pes.licualicola*: 6 bp, *Pes.pandanicola*: 8 bp); *tef1-α* gene (*Pes.krabiensis*: 15 bp, *Pes.licualicola*: 3 bp, *Pes.pandanicola*: 8 bp); *tub2* gene (*Pes.krabiensis*: 12 bp, *Pes.licualicola*: 8 bp, *Pes.pandanicola*: 17 bp). Moreover, *Pes.shanweiensis* differs from *Pes.krabiensis* in having shorter, but wider conidia (*Pes.shanweiensis*: 17.5–22.5 × 5.5–7.5 μm vs. *Pes.krabiensis*: 19–25 × 4–6 μm), longer appendages (*Pes.shanweiensis*: 3–6.5 μm vs. *Pes.krabiensis*: 2–4 μm); *Pes.shanweiensis* differs from *Pes.licualicola* and *Pes.pandanicola* in having larger conidia (*Pes.shanweiensis*:17.5–22.5 × 5.5–7.5 μm vs. *Pes.licualicola*: 16–20 × 3–5 μm, *Pes.pandanicola*: 13–18 × 2.5–4.5 μm). Based on both phylogenetic and morphological evidence, we propose the recognition of *Pes.shanweiensis* as a novel species.

#### ﻿ Pestalotiopsis thunbergii

Taxon classificationAnimaliaAmphisphaerialesSporocadaceae

Q.C. Wang & X.D. Zhou sp. nov.

18BD40FC-DE4B-5F44-B675-E044C9B7A6F8

MB858314

Fig. 13

##### Etymology.

Named after the host, *Pinusthunbergii*.

##### Typus.

CHINA • Shandong Province, Qingdao City, Jimo District, Tianheng Island Resort, 36°25'2"N, 120°51'37"E, on diseased needle of *Pinusthunbergii*, 9 August 2023, *Quanchao Wang, Guoqing Li, Feifei Liu & Runlei Chang* (holotype designated here HMAS 353938, dried culture prepared from ZXD583; ex-holotype culture ZXD583 = CFCC 72589).

##### Description.

Sexual state not seen. ***Conidiomata*** in culture sporodochial, saucer-shaped, scattered or gregarious, superficial to immersed, shining, releasing black conidial masses on the surface. ***Conidiophores*** branched, subcylindrical, hyaline to light brown, indistinct, often reduced to conidiogenous cells. ***Conidiogenous*** cells cylindrical or ampulliform, hyaline, smooth-walled, solitary to aggregated, (4.5–)5.5–10.5(–15) × (1.5–)2–3(–3.5) μm (x ± SD = 8 ± 2.4 × 2.5 ± 0.6 μm). ***Conidia*** fusoid, ellipsoid, smooth, slightly constricted at the septa, 4-septate, (20–)23–27.5(–34) × (6–)6.5–8(–10) μm (x ± SD = 25.1 ± 2.3 × 7.3 ± 0.9 μm); three median cells doliiform, wall verruculose, concolourous, (13.5–)15.5–17.5(–19) μm (x ± SD = 16.4 ± 1 μm) long; second cell from the base (4.5–)5–6(–6.5) μm (x ± SD = 5.7 ± 0.5 μm) long; third cell (4.5–)5–6(–6.5) μm (x ± SD = 5.4 ± 0.5 μm); fourth cell (3.5–)4.5–6(–6.5) μm (x ± SD = 5.3 ± 0.7 μm); apical cell conic with an acute apex, thin- and smooth-walled, hyaline to pale brown, (1–)2.5–4.5(–5) μm (x ± SD = 3.5 ± 1.1 μm) long, with 2–4 tubular appendages (mostly three); apical appendages arising from an apical crest, unbranched, filiform, bent, (6.5–)12–21.5(–29.5) μm (x ± SD = 16.8 ± 4.7 μm); basal cell obconic with a truncate base, thin-walled, hyaline or pale brown, (2.5–)3.5–6(–8.5) μm (x ± SD = 4.7 ± 1.2 μm) long, with one appendage; basal appendage tubular, centric, unbranched, occasionally swollen at the tip, (3.5–)4.5–6.5(–8) μm (x ± SD = 5.4 ± 1.1 μm) long.

**Figure 13.**
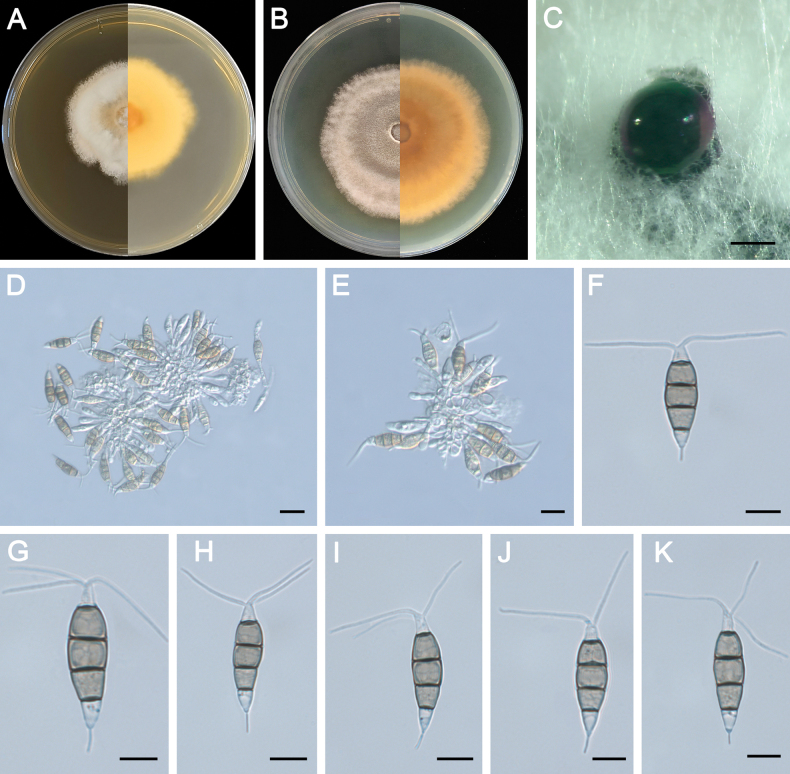
Morphology of *Pestalotiopsisthunbergii* (ZXD583). **A** Colony on MEA after 10 days at 25°C; **B** colony on PDA after 10 days at 25°C; **C** conidiomata formed on MEA; **D, E** conidiogenous cells giving rise to conidia; **F–K** conidia. Scale bars: 500 μm (**C**); 20 μm (**D–G**).

##### Culture characteristics.

Colonies forming abundant flocculent aerial mycelium at the edges on PDA at 25°C, with regular margins, white, the centre of the back of the colony orange, forming black conidiomata with black conidial masses. Optimal growth temperature at 25°C, no growth at 5°C and 35°C; after 10 d, colonies at 10, 15, 20, 25 and 30°C reached 35.1, 41.5, 81.4, 87.8 and 29.4 mm, respectively (Fig. 8).

##### Additional materials examined.

CHINA • Zhejiang Province, Jiaxing City, Pinghu County, Jiulongshan Forest Park, 30°36'10"N, 121°08'27"E, on diseased needle of *Pinusmassoniana*, 15 May 2023, *Quanchao Wang, Guiyong Cao* (culture ZXD204); • Shandong Province, Qingdao City, Jimo District, 36°25'2"N, 120°51'37"E, on diseased needle of *Pinusthunbergii*, 9 August 2023, *Quanchao Wang, Guoqing Li, Feifei Liu & Runlei Chang* (cultures ZXD524, ZXD527, ZXD531, ZXD548, ZXD558, ZXD566, ZXD568, ZXD569, ZXD575, ZXD576, ZXD581).

##### Notes.

*Pestalotiopsisthunbergii* formed a well-supported independent clade (ML/BI = 100/1) from *Pes.ningboensis* (Fig. 3). The differences between *Pes.thunbergii* and *Pes.ningboensis* have been mentioned above (see *Pes.ningboensis*).

#### ﻿ Pestalotiopsis wenzhouensis

Taxon classificationAnimaliaAmphisphaerialesSporocadaceae

Q.C. Wang & X.D. Zhou sp. nov.

982D6A23-6601-50B9-9BDA-BB6C239EA353

MB858316

Fig. 14

##### Etymology.

Named after the collection site of the type specimen, Wenzhou City.

##### Typus.

CHINA • Zhejiang Province, Wenzhou City, Cangnan County, Dayu Town, 27°22'37"N, 120°36'48"E, on diseased needle of *Pinusmassoniana*, 6 May 2023, *Quanchao Wang, Guiyong Cao* (holotype designated here HMAS 353936, dried culture prepared from ZXD64; ex-holotype culture ZXD64 = CFCC 72587).

##### Description.

Sexual state not seen. ***Conidiomata*** in culture sporodochial, saucer-shaped, scattered or gregarious, superficial to immersed, shining, releasing black conidial masses on the surface. ***Conidiophores*** branched, subcylindrical, hyaline to light brown, indistinct, often reduced to conidiogenous cells. ***Conidiogenous cells*** cylindrical or ampulliform, hyaline, smooth-walled, solitary to aggregated, (7–)8–13.5(–18) × (2–)2.5–4(–5) μm (x ± SD = 10.8 ± 2.6 × 3.4 ± 0.7 μm). ***Conidia*** fusoid, ellipsoid, smooth, slightly constricted at the septa, four-septate, (20–)21.5–25(–27.5) × 7–8 μm (x ± SD = 23.2 ± 1.6 × 7.4 ± 0.4 μm); three median cells doliiform, wall verruculose, concolourous, (12.5–)13.5–15.5(–17.5) μm (x ± SD = 14.6 ± 1.1 μm) long; second cell from the base (4–)4.5–5.5(–6) μm (x ± SD = 5 ± 0.4 μm) long; third cell (3.5–)4–5(–5.5) μm (x ± SD = 4.6 ± 0.4 μm); fourth cell (3–)4–5.5(–6.5) μm (x ± SD = 4.9 ± 0.7 μm); apical cell conic with an acute apex, thin- and smooth-walled, hyaline to pale brown, (2.5–)3–4(–4.5) μm (x ± SD = 3.5 ± 0.5 μm) long, with 2–4 tubular appendages (mostly three); apical appendages arising from an apical crest, unbranched, filiform, bent, (7–)9.5–15(–19.5) μm (x ± SD = 12.1 ± 2.7 μm); basal cell obconic with a truncate base, thin-walled, hyaline or pale brown, (3.5–)4.5–5.5(–6) μm (x ± SD = 5 ± 0.6 μm) long, with one appendage; basal appendage tubular, centric, unbranched, occasionally swollen at the tip, (2.5–)3–4.5(–6) μm (x ± SD = 3.9 ± 0.7 μm) long.

**Figure 14.**
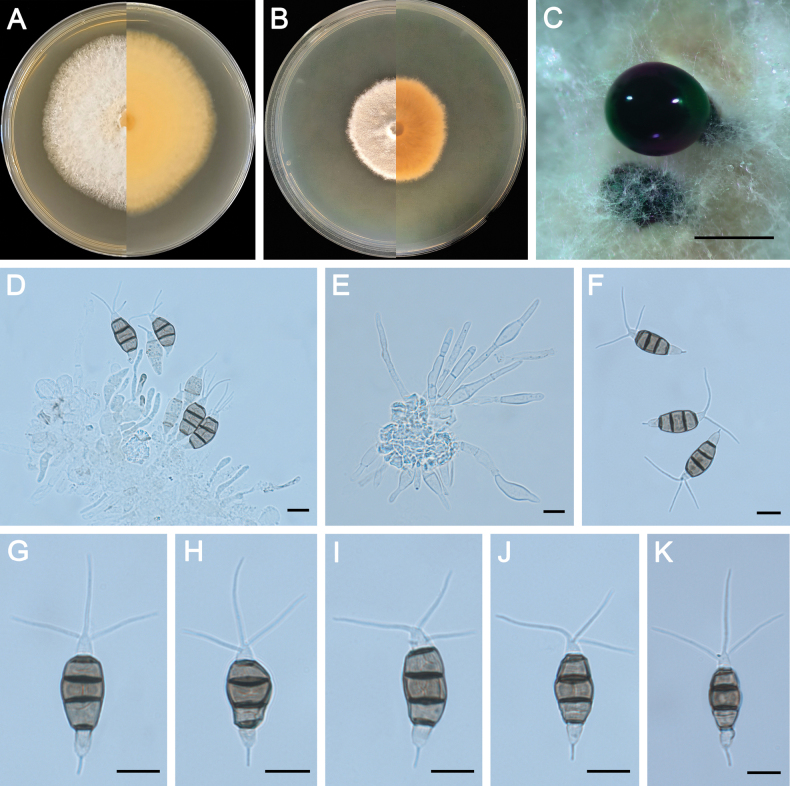
Morphology of *Pestalotiopsiswenzhouensis* (ZXD966). **A** Colony on MEA after 10 days at 25°C; **B** colony on PDA after 10 days at 25°C; **C** conidiomata formed on MEA; **D, E** conidiogenous cells giving rise to conidia; **F–K** conidia. Scale bars: 500 μm (**C**); 20 μm (**D–G**).

##### Culture characteristics.

Colonies exhibiting flocculent aerial mycelium on PDA at 25°C, with regular margins at the edges, white to isabelline, the back colony light orange, forming black conidiomata with black conidial masses. Optimal growth temperature at 25°C, no growth at 5°C and 35°C; after 10 d, colonies at 10, 15, 20, 25 and 30°C reached 31.9, 48.2, 74.5, 88.8 and 57.6 mm, respectively (Fig. 8).

##### Additional materials examined.

CHINA • Zhejiang Province, Wenzhou City, Cangnan County, Dayu Town, 27°22'37"N, 120°36'48"E, on diseased needle of *Pinusmassoniana*, 6 May 2023, *Quanchao Wang, Guiyong Cao* (cultures ZXD79, ZXD86); • Guangdong Province, Jiangmen City, Taishan County, Chixi Town, 21°53'10"N, 112°57'0"E, on diseased needle of *Pinusmassoniana*, 28 August 2023, *Quanchao Wang, Guoqing Li, Feifei Liu & Yuhua Liang* (cultures ZXD957, ZXD958, ZXD966, ZXD970, ZXD973, ZXD974).

##### Notes.

*Pestalotiopsiswenzhouensis* forms a well-supported independent clade (ML/BI = 100/0.99) and is phylogenetically distinct from *Pes.abietis* (Fig. 3). The phylogenetic differentiation between these two species was supported by nucleotide variations in ITS (3 bp), *tub2* (3 bp) and *tef1-α* (1 bp). Morphologically, *Pes.wenzhouensis* can be distinguished from *Pes.abietis* by its larger conidia (*Pes.wenzhouensis*: 20–27.5 × 7–8 μm vs. *Pes.abietis*: 19.9–31.2 × 5.8–8 μm). Furthermore, *Pes.wenzhouensis* exhibits more and longer apical appendages (*Pes.wenzhouensis*: 7–19.5 μm, n = 2–4; *Pes.abietis*: 2.4–6 μm, n = 1–3) and basal appendage (*Pes.wenzhouensis*: 2.5–6 μm vs. *Pes.abietis*: 1.3–5.2 μm). Based on both phylogenetic and morphological evidence, we propose the recognition of *Pes.wenzhouensis* as a novel species.

### ﻿Prevalence

**Figure 15.**
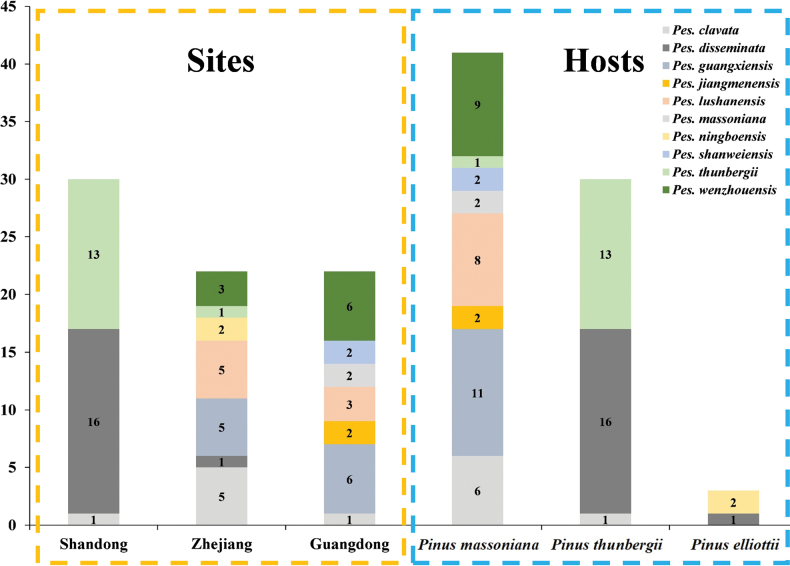
Isolates of *Pestalotiopsis* strains from different sites and hosts.

In this study, the strains of *Pestalotiopsis* were collected from Shandong, Zhejiang and Guangdong Provinces. Amongst these regions, Shandong exhibited the highest frequency of isolation (40.6%, three species), followed by Zhejiang (29.7%, seven species) and Guangdong (29.7%, seven species) (Fig. 15). The host association revealed that *P.massoniana* accounted for the largest number of strains and species (55.4%, eight species), followed by *P.thunbergii* (40.5%, three species) and *P.elliottii* (4.1%, two species). These results might be biased considering that all samples collected from Shandong were derived from *P.thunbergii*, while those obtained from Zhejiang and Guangdong primarily originated from *P.massoniana*. Additionally, fewer samples were collected on *P.elliottii* due to its lower disease incidence rate compared to other *Pinus* species (Fig. 15).

## ﻿Discussion

*Pestalotiopsis* species exhibit a global distribution and demonstrate a wide range of hosts. The Global Biodiversity Information Facility (https://www.gbif.org/, accessed on 24 November 2024) displays its 12195 records and the abundance of the top five countries is USA, India, Australia, Brazil and China. In China, Zhu et al. (1991) described seven novel *Pestalotiopsis* species and Zhao and Li (1995) reported the discovery of 34 *Pestalotiopsis* species in Yunnan, based on morphological characteristics. The combination of morphology and gene sequencing comparison has further boosted the work of *Pestalotiopsis* taxonomy. In 2007, 19 *Pestalotiopsis* species were identified from *P.armandii* and seven species from *Ribes* plants (Hu et al. 2007). Maharachchikumbura et al. (2012) discovered another 23 species including 14 novel ones in China. Fifteen *Pestalotiopsis* species including eight novel ones were obtained from *Camellia* plants, while 16 species (10 novel species) from 20 *Fagaceae* hosts (Liu et al. 2017; Jiang et al. 2022c) and 14 novel species within the genus *Pestalotiopsis* were identified by Razaghi et al. (2024). Here, we reported that 10 *Pestalotiopsis* species (six novel species) were obtained from *P.elliottii*, *P.massoniana* and *P.thunbergii*. These indicate that the diversity of *Pestalotiopsis* species in China is high and deserves further continuous exploration.

Many *Pestalotiopsis* species are pathogenic and cause leaf spots, root rot or fruit rot on various plants (Sati and Belwal 2005; Keith 2008; Chen et al. 2012; Razaghi et al. 2024). In China, pine red blight caused by *Pes.funerea* on *P.massoniana* was first recorded in 1974 in Sichuan Province (Qiu et al. 1980) and subsequently documented in Heilongjiang, Shandong, Jiangsu and Guangdong Provinces (Xu et al. 2017; Chen et al. 2020; Li et al. 2024). The disease also has been reported from other parts of the world such as Portugal, Spain and Tunisia (Silva et al. 2020; Hlaiem et al. 2022; Monteiro et al. 2022). Until now, species of *Pes.biciliata*, *Pes.crina*, *Pes.funerea*, *Pes.jiangsuensis*, *Pes.neglecta*, *Pes.pini*, *Pes.rosea* and *Pes.trachicarpicola* have been considered responsible for pine blight (Xu et al. 2017; Chen et al. 2020; Silva et al. 2020; Qi et al. 2021; Hlaiem et al. 2022; Han et al. 2024; Li et al. 2024). However, other *Pestalotiopsis* species, such as *Pes.algeriensis*, *Pes.carveri*, *Pes.cocculi*, *Pes.disseminata*, *Pes.lawsoniae*, *Pes.lespedeza* and *Pes.neglecta*, are endophytic (Hu et al. 2007; Liu et al. 2013). The *Pestalotiopsis* strains obtained from this study were all isolated from pine needles exhibiting typical symptoms of pine red blight. Their possible pathogenicity and potential impact will be evaluated.

*Pinusmassoniana* is the most widely distributed and afforested timber tree species in China (Lu et al. 2022). Previous studies disclosed that species of *Pes.funerea* and *Pes.jiangsuensis* cause the needle blight on *P.massoniana* (Qiu et al. 1980; Li et al. 2024). Liu et al. (2013) reported that six *Pestalotiopsis* species are endophytic to *P.massoniana*. Here, eight *Pestalotiopsis* species were found on *P.massoniana* (Fig. 15), including *Pes.clavata*, *Pes.guangxiensis*, *Pes.jiangmenensis*, *Pes.lushanensis*, *Pes.massoniana*, *Pes.shanweiensis*, *Pes.thunbergii* and *Pes.wenzhouensis*. Notably, apart from *Pes.lushanensis*, the other seven *Pestalotiopsis* species have not been recorded from pines. Our findings contribute to a further understanding of *Pestalotiopsis* species inhabiting *P.massoniana*, the most important pine species in China.

Temperature plays a crucial role affecting the growth, sporulation and infection of *Pestalotiopsis* species (Das et al. 2010; Fovo et al. 2017). In this study, the samples were collected from three distinct climatic zones, namely Shandong (temperate monsoon climate), Zhejiang (subtropical monsoon climate) and Guangdong (tropical monsoon climate). Growth experiments were conducted at different temperatures on six novel species. The results revealed that all six species demonstrated optimal growth conditions at 25°C (Fig. 7). However, growth variations amongst different species emerged at 30°C. Two species, *Pes.jiangmenensis* and *Pes.massoniana*, isolated primarily from the Guangdong Region exhibited sustained high colony growth rates even at 30°C. Conversely, *Pes.thunbergii* colony growth rate reached its lowest level at 30°C, which was predominantly isolated from the Shandong Region. The findings suggest that the different *Pestalotiopsis* species may have developed their own distinct temperature adaptation mechanisms through extensive evolutionary processes in diverse geographical environments.

## ﻿Conclusions

This study represents the most comprehensive survey on *Pestalotiopsis* inhabiting pines from different climate zones in China and enhances our knowledge on this group of fungi. In total, ten *Pestalotiopsis* species were obtained, including six new to science which are described here. The results further revealed that *Pestalotiopsis* species exhibit distinct host preferences, which appear to be influenced by climatic conditions. Considering the current study confined to three important pine species — *P.elliottii*, *P.massoniana* and *P.thunbergii* and the vast and ecologically diverse landscapes distributed by various pine species there, it is highly probable that numerous *Pestalotiopsis* species remain to be discovered.

## Supplementary Material

XML Treatment for Pestalotiopsis clavata

XML Treatment for Pestalotiopsis disseminata

XML Treatment for Pestalotiopsis guangxiensis

XML Treatment for Pestalotiopsis jiangmenensis

XML Treatment for Pestalotiopsis lushanensis

XML Treatment for Pestalotiopsis massoniana

XML Treatment for Pestalotiopsis ningboensis

XML Treatment for Pestalotiopsis shanweiensis

XML Treatment for Pestalotiopsis thunbergii

XML Treatment for Pestalotiopsis wenzhouensis

## ﻿Additional information

### Adherence to national and international regulations

All the fungal strains used in this study have been legally obtained, respecting the Convention on Biological Diversity (Rio Convention).

### Author ORCIDs

QuanChao Wang https://orcid.org/0009-0007-9170-0326

ZhaoJie Zhan https://orcid.org/0009-0003-3680-377X

Adil Sattar https://orcid.org/0009-0009-2709-1006

HaoNan Wang https://orcid.org/0000-0002-9471-8886

LiFeng Zhou https://orcid.org/0000-0003-4922-4710

Lori Eckhardt https://orcid.org/0000-0002-3270-8199

GuoQing Li https://orcid.org/0009-0001-0014-2653

FeiFei Liu https://orcid.org/0000-0002-9820-1323

HuaChao Xu https://orcid.org/0009-0006-6066-3254

XuDong Zhou https://orcid.org/0000-0002-9580-9093

### Data availability

All sequences generated during this study have been submitted to GenBank.

## Supplementary materials

Supplementary material 1
*Pestalotiopsis* ITS
Data type fas Explanation note The ITS (Internal Transcribed Spacer) sequence alignment data of *Pestalotiopsis*. https://binary.pensoft.net/file/1349459This dataset is made available under the Open Database License (http://opendatacommons.org/licenses/odbl/1.0/). The Open Database License (ODbL) is a license agreement intended to allow users to freely share, modify, and use this Dataset while maintaining this same freedom for others, provided that the original source and author(s) are credited.QuanChao Wang, XuDong Zhou

Supplementary material 2 *Pestalotiopsis* TEF Data type fas Explanation note The TEF (translation elongation factor 1-alpha) sequence alignment data of *Pestalotiopsis*. https://binary.pensoft.net/file/1349460This dataset is made available under the Open Database License (http://opendatacommons.org/licenses/odbl/1.0/). The Open Database License (ODbL) is a license agreement intended to allow users to freely share, modify, and use this Dataset while maintaining this same freedom for others, provided that the original source and author(s) are credited.QuanChao Wang, XuDong Zhou

Supplementary material 3 *Pestalotiopsis* TUB Data type fas Explanation note The TUB (partial β-tubulin) sequence alignment data of *Pestalotiopsis*. https://binary.pensoft.net/file/1349461This dataset is made available under the Open Database License (http://opendatacommons.org/licenses/odbl/1.0/). The Open Database License (ODbL) is a license agreement intended to allow users to freely share, modify, and use this Dataset while maintaining this same freedom for others, provided that the original source and author(s) are credited.QuanChao Wang, XuDong Zhou

Supplementary material 4 Phylogenetic tree of *Pestalotiopsis* Data type tre Explanation note Phylogenetic trees based on Maximum Likelihood (ML) analyses from the multi-gene alignment (ITS, *tef-1α* and *tub2*) for *Pestalotiopsis*. https://binary.pensoft.net/file/1349462This dataset is made available under the Open Database License (http://opendatacommons.org/licenses/odbl/1.0/). The Open Database License (ODbL) is a license agreement intended to allow users to freely share, modify, and use this Dataset while maintaining this same freedom for others, provided that the original source and author(s) are credited.QuanChao Wang, XuDong Zhou
